# Supplementary material for: Fe-rich X-ray amorphous material records past climate and persistence of water on Mars
Source: Commun Earth Environ. 2024 Jul 7;5(1):364. doi: 10.1038/s43247-024-01495-4 (PMC11227439; doi:10.1038/s43247-024-01495-4)
Supplement: Supplementary file 2 — Supplementary Information [file 43247_2024_1495_MOESM2_ESM.pdf]

Fe-rich X-Ray amorphous material records past climate and persistence of water on Mars

SUPPLEMENTARY INFORMATION

Anthony D. Feldman[1,a]*, Elisabeth M. Hausrath[1], Elizabeth B. Rampe[2], Oliver Tschauner[1], Valerie Tu[3], Tanya S. Peretyazhko[3], Christopher DeFelice[1,b], Thomas Sharp[4]

[1.]Department of Geosciences, University of Nevada Las Vegas, Las Vegas, NV, USA. [2.]Astromaterials Research and Exploration Science Division, NASA Johnson Space Center, Houston, TX, USA. [3.]Jacobs Technology, NASA Johnson Space Center, Houston, TX, USA. [4.]School of Earth and Space Exploration, Arizona State University, Tempe, AZ, USA.

[a.]Present Address: Desert Research Institute, Las Vegas, NV, USA.

[b.]Present Address: Pacific Northwest National Laboratory, Richland, Washington, USA.

***Corresponding author (anthony.feldman@dri.edu)**

Table of Contents

S1 Supplementary Discussion 2

S1.1 Sampling Site Locations 2

S1.2. Comparison of 1 hour and 3 Day Cation Saturation Procedure 4

S1.3 Reference D-Spacings For Oriented Mount Phase ID and Rietveld Refinement Structure Files 5

S1.4 Labelled XRD Patterns of Randomly Oriented Bulk Soil and Clay-Size Fraction Material and Oriented Clay-Size Fraction Material 6

S1.5 Rietveld Refinements of 20 wt. % α-Al_2O_3 spiked Clay-Size Fraction Samples 22

S1.6 Additional Transmission Electron Microscopy Results 34

S1.7 Hydroxylamine Hydrochloride Extractable Si 35

S1.8 Dithionite Reducible Fe 36

S1.9 Hydroxylamine and Dithionite Selective Dissolution Results Plotted by Depth 37

S1.10 Pyrophosphate Extractable Fe 38

S1.11 Soil Field Descriptions, Soil pH, and Soil Loss on Ignition at 550 ^{o}C 39

S2 Supplementary References 41

S1 Supplementary Discussion

S1.1 Sampling Site Locations

Table S1 – GPS locations in decimal coordinates and elevations of soil pit sampling sites

Sampling Site	Latitude	Longitude	Elevation
**Klamath Mountains**			
Eunice Bluff	41.339083	-122.587389	2106 m
Deadfall Lake	41.314510	-122.501086	2215 m
Swift Creek Late	41.004483	-122.839969	1570 m
Swift Creek Middle	40.986972	-122.820828	1355 m
String Bean Creek	40.367500	-123.073033	1174 m
**Tablelands**			
Devil's Punchbowl	49.480056	-58.016667	292 m
Winterhouse Gulch Canyon	49.456124	-57.962366	313 m
Winterhouse Gulch Mouth	49.464395	-57.959659	261 m
Trout River Gulch	49.479041	-57.980459	155 m
**Pickhandle Gulch**			
Summit	38.1481778	-118.0678572	1850 m
Footslope 1	38.1485219	-118.0679719	1840 m
Footslope 2	38.1486440	-118.067703	1837 m

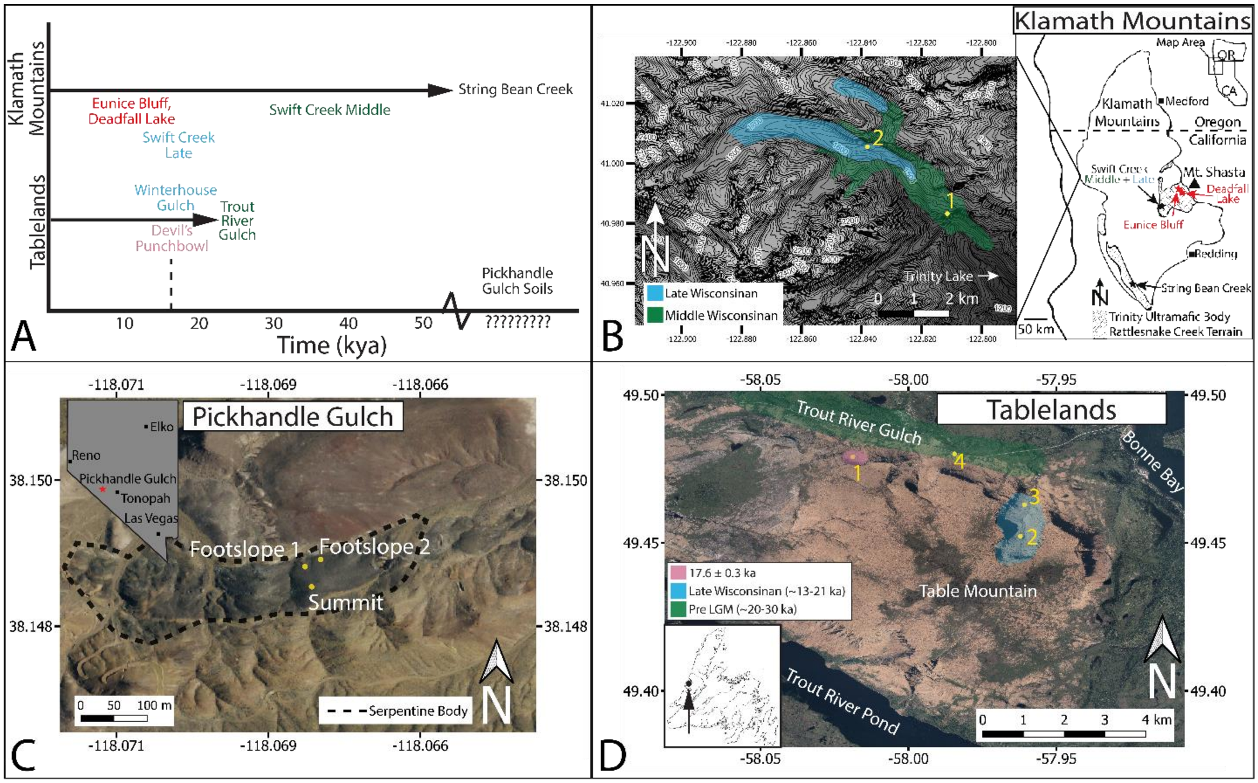

Figure S1 – Age constraints and locations of soil sampling sites. A) Age constraints for soil sampling locations within the Klamath Mountains and Newfoundland Tablelands. Soils within the Trinity Ultramafic Body are estimated to have begun development roughly 12.1 kya as paleolake records suggest warming at that time within the Trinity Alps[1] and reconstructions of glacial activity suggest deglaciation concurrently with the end of the Wisconsin glacial epoch [2]. Recent Cl-36 dates confirm deglaciation of high-altitude cirques in the Klamath Mountain around 12.1 kya [3]. The String Bean Creek site within the Rattlesnake Creek terrane has not been dated. However, to our knowledge no studies have documented glacial landforms within the Rattlesnake Creek Terrane. Within the Tablelands, the youngest sampled soil is developing within the Devil's Punchbowl cirque, where the end moraine was Cl-36 dated to 17.6 ka [4]. The Winterhouse Gulch valley is not cosmogenically dated but was likely deglaciated following the late Wisconsinan glacial period at the close of the Pleistocene[4] around ~13-21 ka. The Trout River Gulch soil is likely to have been exposed prior to the last glacial maximum[4], indicating soil development initiated sometime prior to 20 kya[5]. B) Locations of sampling sites in the Klamath Mountains marked on a contour map of the Swift Creek sampling area, numbered field sites are 1. Swift Creek Late and 2. Swift Creek Middle. C) Locations of sampling sites at Pickhandle Gulch in Nevada. D) Sampling sites at the Tablelands in Newfoundland, Canada. Field sites are 1. Devil's Punchbowl, 2. Winterhouse Gulch Canyon, 3. Winterhouse Gulch Mouth and 4. Trout River Gulch. Maps were created using QGIS software with orthoimagery and 1m DEM files from the USDA NRCS GeoSpatialDataGateway for areas within the United States and orthophotos from the Newfoundland and Labrador Fisheries, Forestry, and Agriculture GIS and Mapping Division for imagery from Canada. The inset in panel B is modified from Baumeister et al. (2015)[6].

S1.2 XRD analyses of oriented clay-size fraction mounts after 1 hour and 3 Day Cation Saturation, and then again after solvation by ethylene glycol vapor

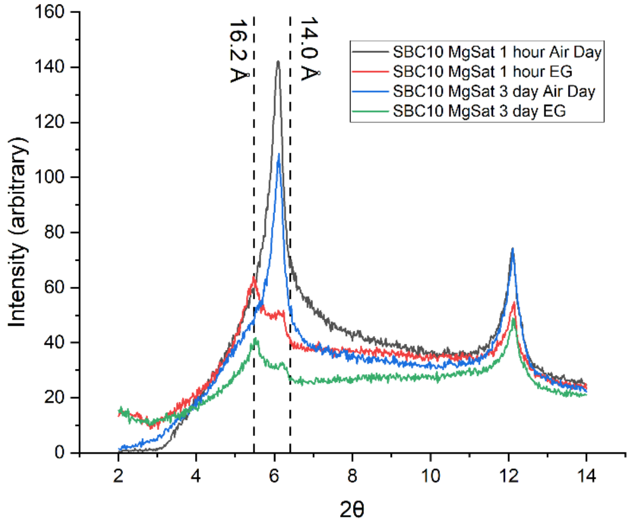

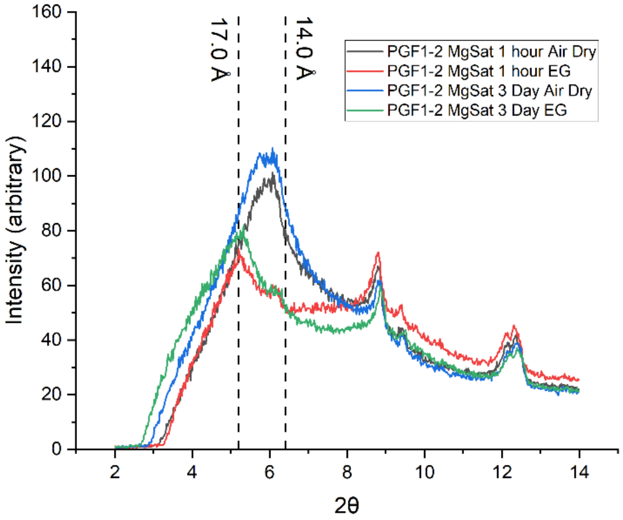

Figure S2 – Comparison of 3-day and 1-hour cation saturated oriented mount XRD patterns followed by XRD analysis after ethylene glycolation. XRD patterns (Cu K-alpha source) of oriented mounts of the clay-size fraction from 90-100 cm in the String Bean Creek soil (left) and 4-10 cm in the Footslope 1 soil at Pickhandle Gulch (right). Images show oriented mounts that were Mg^{2+} cation saturated for either 1 hour or 3 days (72 hours) and then air dried and measured by XRD, followed by solvation of the same cation saturated oriented mounts with ethylene glycol vapor for 12 hours at 60-70°C and measurement by XRD. Peak locations in both air dried and ethylene glycol vapor solvated samples are identical between the 1 hour and 3-day treatments, suggesting that a 1-hour treatment achieves similar cation saturation to longer procedures and a 3 day treatment is not required.

S1.3 Reference D-Spacings For Oriented Mount Phase ID and Rietveld Refinement Structure Files

Table S2 – Phyllosilicate d-spacings for oriented clay mount XRD pattern analysis

Mineral	Reference Plane	d-spacing (Å)*	Oriented Mount Treatments
Serpentine	001	7.3	All
Talc	001	9.4	All
Chlorite	001	14	All
	002	7.0-7.1	All
Muscovite	001	10	All
Low Charge Smectite	001	~15	Mg-saturation + Air Drying
		~16-18	Mg-Saturation + Ethylene Glycol Solvation
		~10	K-Saturation + Heating to 550 °C
High-Charge Smectite / Vermiculite	001	~14	Mg-Saturation + Air Drying
		~14-14.5	Mg-Saturation + Ethylene Glycol Solvation
		~10	K-Saturation + Heating to 550 °C
Interstratified Clays**	001	~24+	All

* Characteristic d-spacings for phyllosilicates[7–16].

** Mixed phase phyllosilicates exhibit variable d-spacing based on variations in layer stacking that result in broad peaks/humps at low angles 2θ.

Table S3 – Origins of structure files utilized in clay-size fraction Rietveld refinement

Mineral	Source	BGMN File or COD ID
Amphibole (Tremolite)	Crystallography Open Database	COD 9013319
Calcite	Crystallography Open Database	COD 9016705
Chlorite (Clinochlore)	Crystallography Open Database	COD 9013855
Corundum	Crystallography Open Database	COD 9007498
Goethite	Crystallography Open Database	COD 9003076
Muscovite	Crystallography Open Database	COD 9001962
Plagioclase (Albite)	Crystallography Open Database	COD 9000525
Plagioclase (Anorthite)	Crystallography Open Database	COD 9001261
Quartz	Crystallography Open Database	COD 9013321
Talc	Crystallography Open Database	COD 9008297
Serpentine (Antigorite)*	Crystallography Open Database	COD 9003999
Serpentine (Lizardite)	Crystallography Open Database	COD 9004508
Trioctahedral Smectite (Mg/Fe-Saponite)	Levy et al. (2020)[17]	na
Dioctahedral Smectite (Al/Fe-Montmorillonite)	BGMN Database[18]**	smectitedi2wfix1

***Antigorite was identified by the absence of the asymmetric (020) peaks at ~4.62 Å to 4.57 Å, and included in the Rietveld refinement when identified as present in the XRD analyses. While trace amounts of chrysotile were detected by TEM in four examined soil samples (Figure S44), the presence of chrysotile by XRD could also not be definitively determined, and it is therefore not included in the Rietveld refinements.

**The BGMN database[18] is maintained at http://bgmn.de/download-structures.html and is included with the Profex program[19]

S1.4 Labelled XRD Patterns of Randomly Oriented Parent Material, Bulk Soil, and Clay-Size Fraction Material and Oriented Clay-Size Fraction Material

Observed XRD patterns for parent material, bulk soil, and clay-size fraction material can be found in Supplementary Data 1.

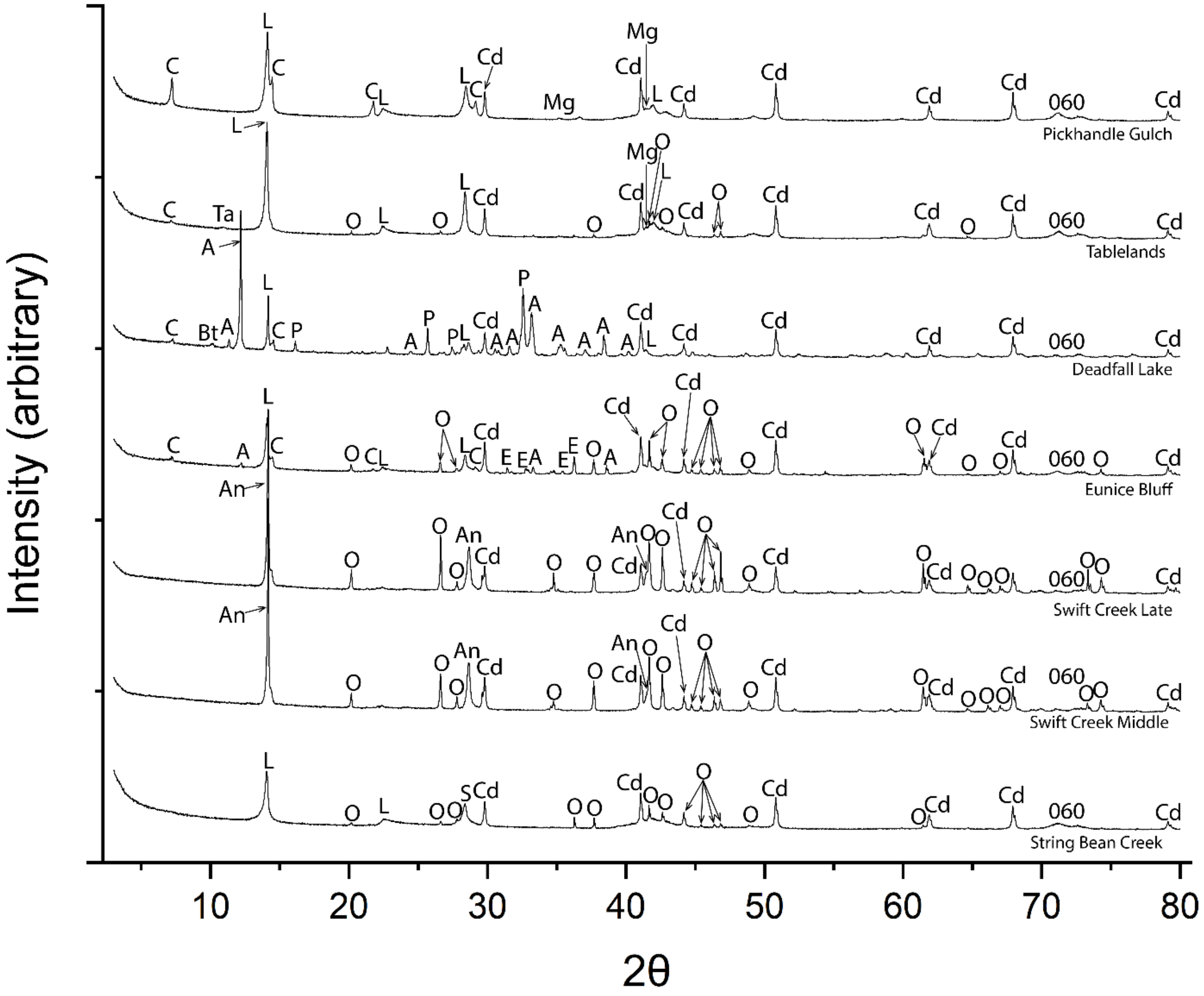

Figure S3 – Randomly oriented XRD patterns (Co K-alpha source) of samples of parent material from each soil pit in the Klamath Mountains, Tablelands, and Pickhandle Gulch sites. Abbreviations: Amphibole (A), Antigorite (An), Biotite (Bt), Chlorite (C), Corundum (Cd), Enstatite (E), Lizardite (L), Olivine (O), Plagioclase (P), Talc (Ta), Tremolite (Tr). Antigorite vs. Lizardite serpentine mineral presence was determined based on the presence (Lizardite) or absence (Antigorite) of an asymmetric 02l peak at ~4.58Å6. Lizardite is present in all parent material samples except the Swift Creek Late and Middle parent material, where the absence of the ~4.58Å peak denotes antigorite presence. Corundum peaks in the clay-size fraction patterns result from spiking for potential Rietveld refinement.

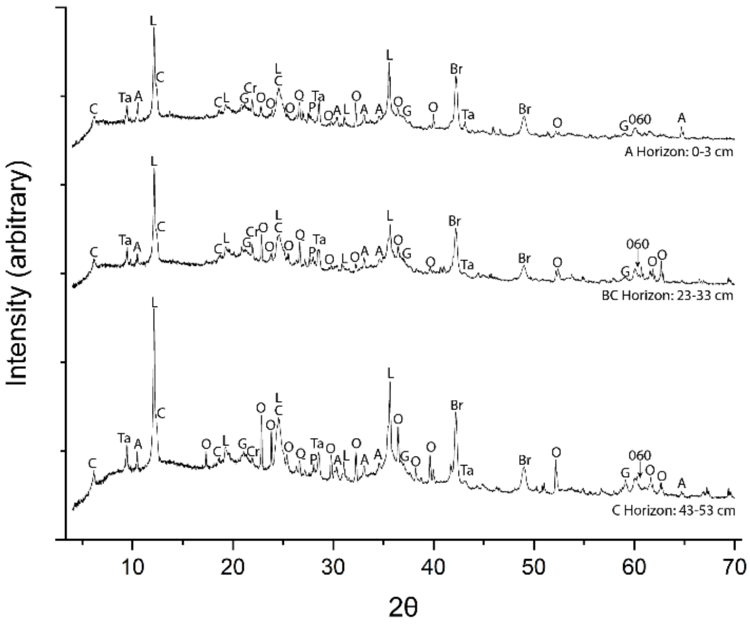

Figure S4 – Labelled XRD patterns (Cu Kα source) from randomly oriented mounts of bulk soil material from the Eunice Bluff soil in the Klamath Mountains. Abbreviations: Amphibole (A), Brass (Br), Chlorite (C), Goethite (G), Lizardite (L), Olivine (O), Quartz (Q), Plagioclase (P), Talc (Ta). 060 peaks associated with phyllosilicates are marked as well. Peaks marked B in the bulk soil patterns result from use of a Brass sample stage.

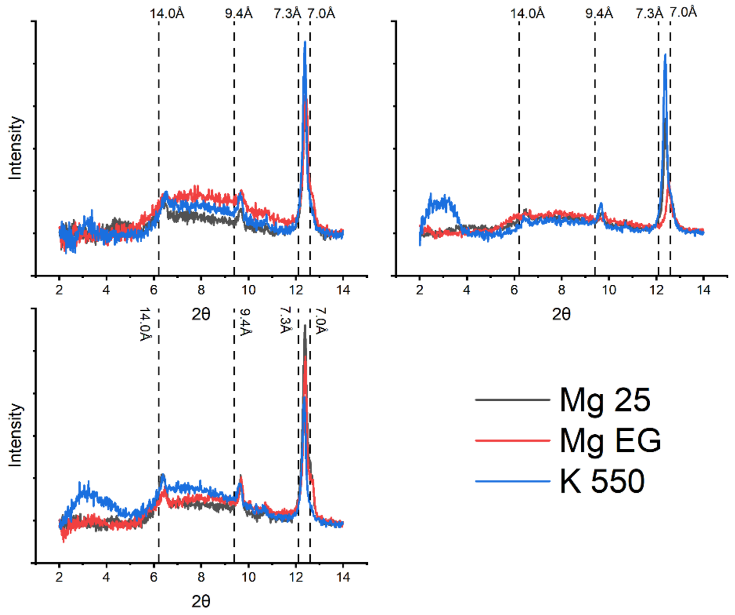

Figure S5 – Eunice Bluff clay-size fraction oriented XRD patterns measured after treatment with Mg saturation followed by air-drying (MG 25), Mg saturation followed by Ethylene glycolation (Mg EG), and K saturation followed by heating at 550 °C (K550) as described in the online methods. Marked d-spacings correspond to phyllosilicate peak ID in Table S2; 7.3 Å for serpentine, 14 Å and 7.0 Å for chlorite, 9.4 Å for talc.

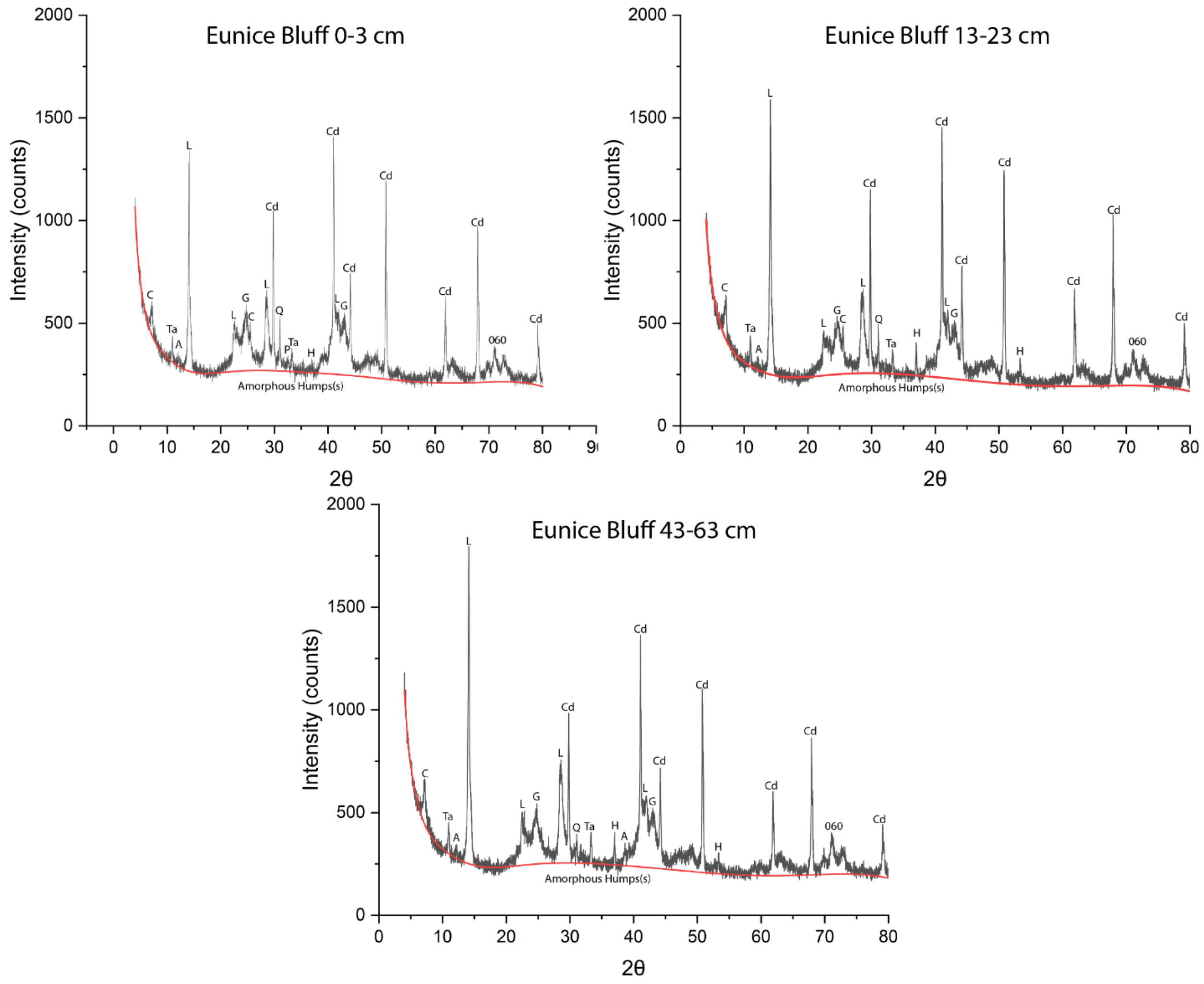

Figure S6 – Labelled XRD patterns (Co Kα source) from randomly oriented mounts of soil clay-size fraction material from the Eunice Bluff soil in the Klamath Mountains. The background plus amorphous determined by Rietveld refinement is given in red. The locations of the amorphous hump(s) are marked. Abbreviations: Amphibole (A), Chlorite (C), Corundum (Cd), Goethite (G), Halite (H), Lizardite (L), Quartz (Q), Plagioclase (P), Talc (Ta). 060 peaks associated with phyllosilicates are marked as well. Corundum peaks in the clay-size fraction result from spiking for Rietveld refinement. Halite peaks represent minor residual presence from the clay-size extraction procedure.

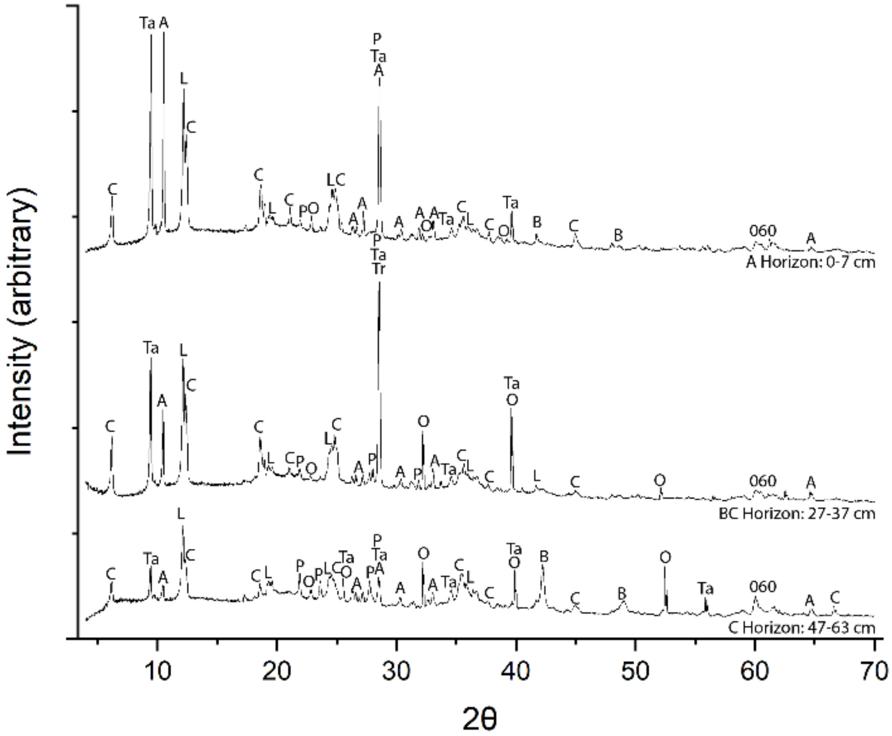

Figure S7 –Labelled XRD patterns (Cu Kα source) from randomly oriented mounts of bulk soil from the Deadfall Lake soil in the Klamath Mountains. 060 peaks associated with phyllosilicates are marked as well. Abbreviations: Brass (B), Chlorite (C), Corundum (Cd), Lizardite (L) Olivine (O), Quartz (Q), Plagioclase (P), Talc (Ta). Peaks marked B result from using a Brass sample stage for XRD analysis.

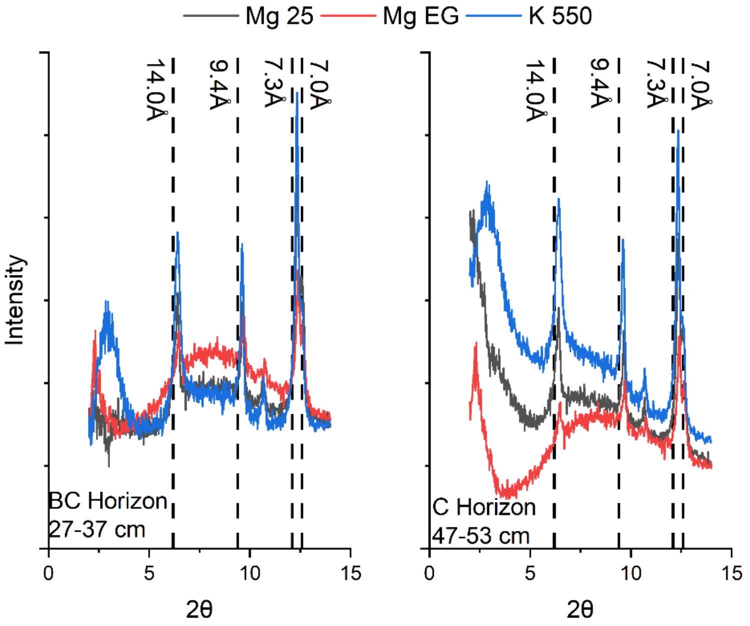

Figure S8 – Deadfall Lake clay-size fraction oriented XRD patterns measured after treatment with Mg saturation followed by air-drying (MG 25), Mg saturation followed by Ethylene glycolation (Mg EG), and K saturation followed by heating at 550 °C (K550) as described in the online methods. Marked d-spacings correspond to phyllosilicate peak IDs in Table S2; 7.3 Å for serpentine, 14 Å and 7.0 Å for chlorite, 9.4 Å for talc.

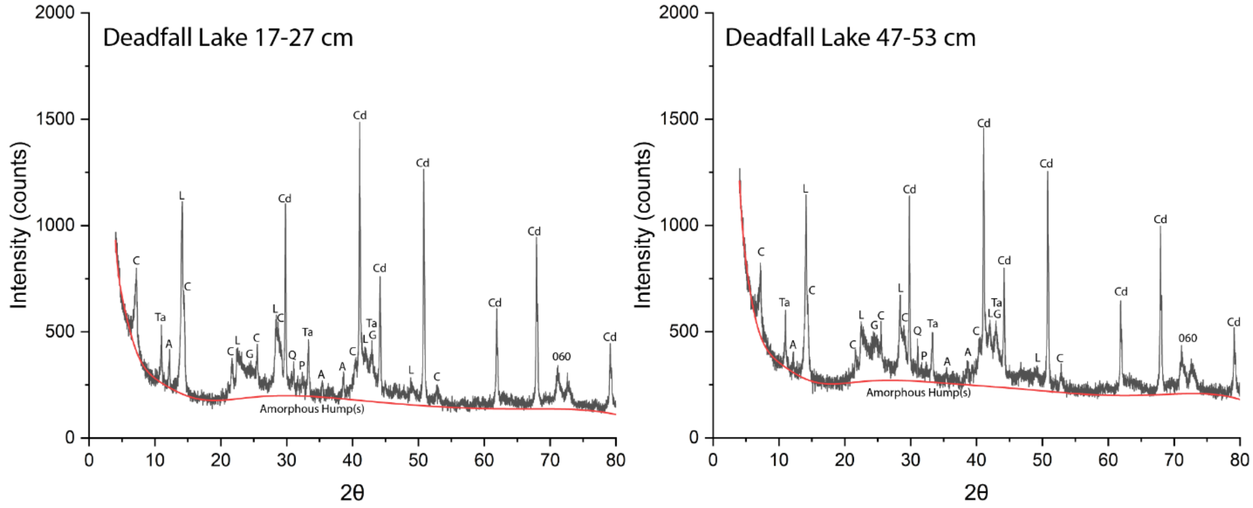

Figure S9 –Labelled XRD patterns (Co Kα source) from randomly oriented mounts of clay-size fraction material from the Deadfall Lake soil in the Klamath Mountains. The background plus amorphous determined by Rietveld refinement is given in red. The locations of the amorphous hump(s) are marked. Abbreviations: Chlorite (C), Corundum (Cd), Lizardite (L) Olivine (O), Quartz (Q), Plagioclase (P), Talc (Ta). 060 peaks associated with phyllosilicates are marked as well. Corundum peaks in the clay-size fraction patterns results from spiking for Rietveld refinement.

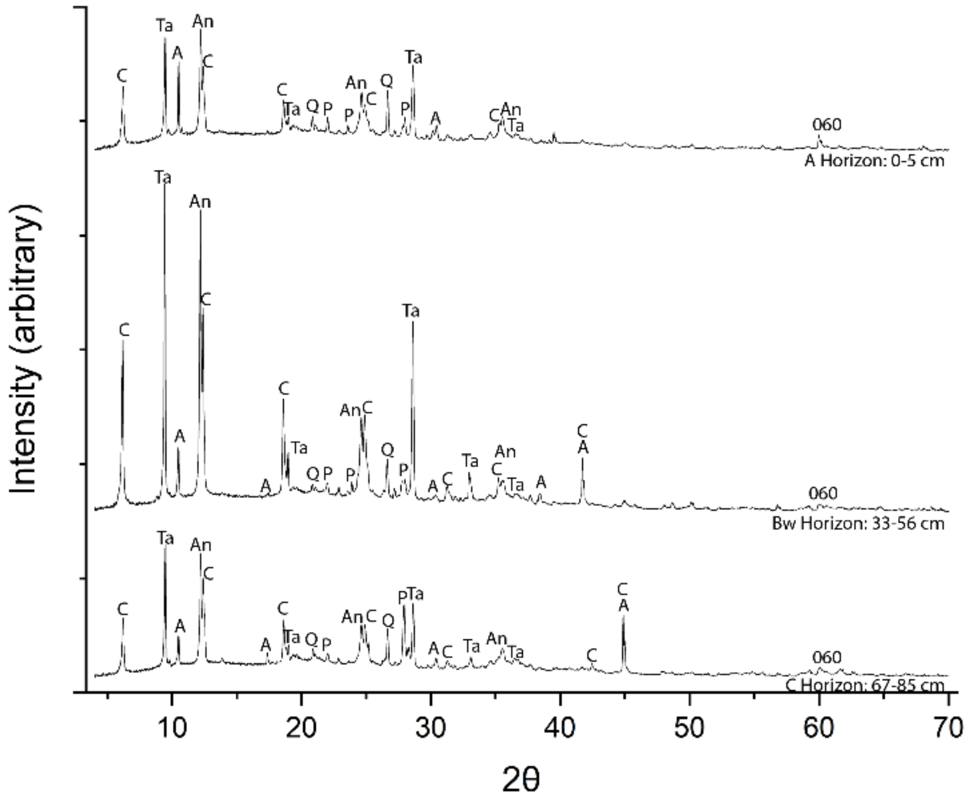

Figure S10 – Labelled XRD patterns (Cu Kα source) from randomly oriented mounts of bulk soil from the Swift Creek Late soil in the Klamath Mountains. 060 peaks associated with phyllosilicates are marked as well. Abbreviations: Amphibole (A), Antigorite (An), Chlorite (C), Quartz (Q), Plagioclase (P), Talc (Ta).

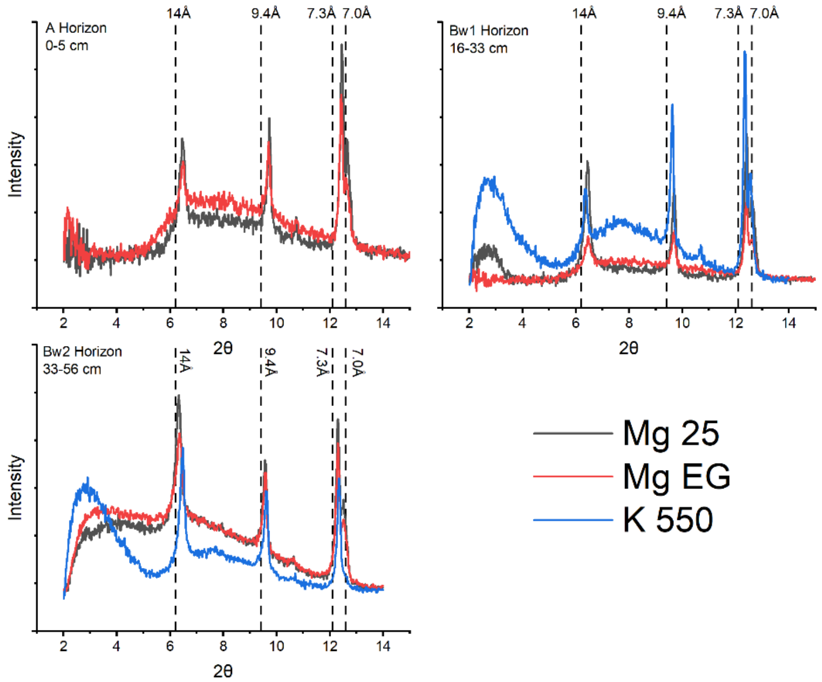

Figure S11 – Swift Creek Late oriented clay-size fraction XRD patterns measured after treatment with Mg saturation followed by air-drying (MG 25), Mg saturation followed by Ethylene glycolation (Mg EG), and K saturation followed by heating at 550 °C (K550) as described in the online methods. Marked d-spacings correspond to phyllosilicate peak ID in table S2; 7.3 Å for serpentine, 14 Å and 7.0 Å for chlorite, 9.4 Å for talc.

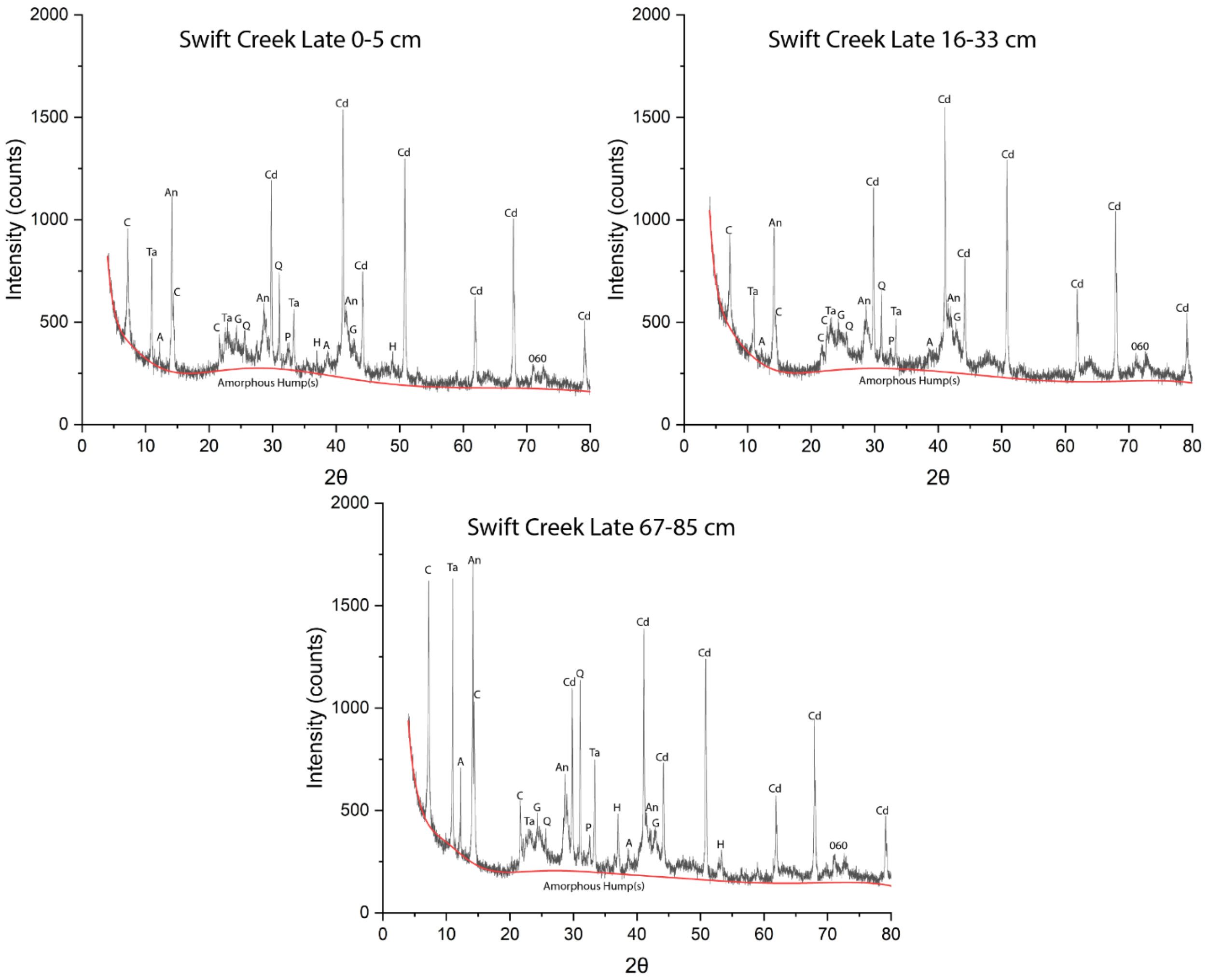

Figure S12: Labelled XRD patterns (Co Kα source) from randomly oriented mounts of soil clay-size fraction material from the Swift Creek Late soil in the Klamath Mountains. The background plus amorphous determined by Rietveld refinement is given in red. The locations of the amorphous hump(s) are marked. Abbreviations: Amphibole (A), Antigorite (An), Chlorite (C), Corundum (Cd), Goethite (G), Halite (H), Quartz (Q), Plagioclase (P), Talc (Ta). 060 peaks associated with phyllosilicates are marked as well. Corundum peaks in the clay-size fraction result from spiking for Rietveld refinement. Halite peaks represent minor residual presence from the clay-size extraction procedure.

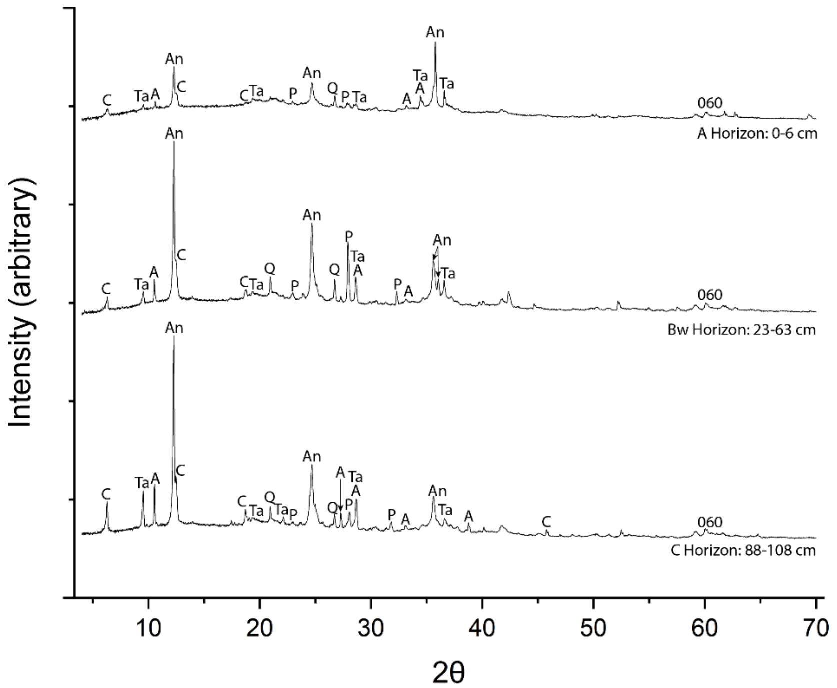

Figure S13 – Labelled XRD patterns (Cu Kα source) from randomly oriented mounts of bulk soil from the Swift Creek Middle soil in the Klamath Mountains. Abbreviations: Amphibole (A), Antigorite (An), Chlorite (C), Quartz (Q), Plagioclase (P), Talc (Ta). 060 peaks associated with phyllosilicates are marked as well.

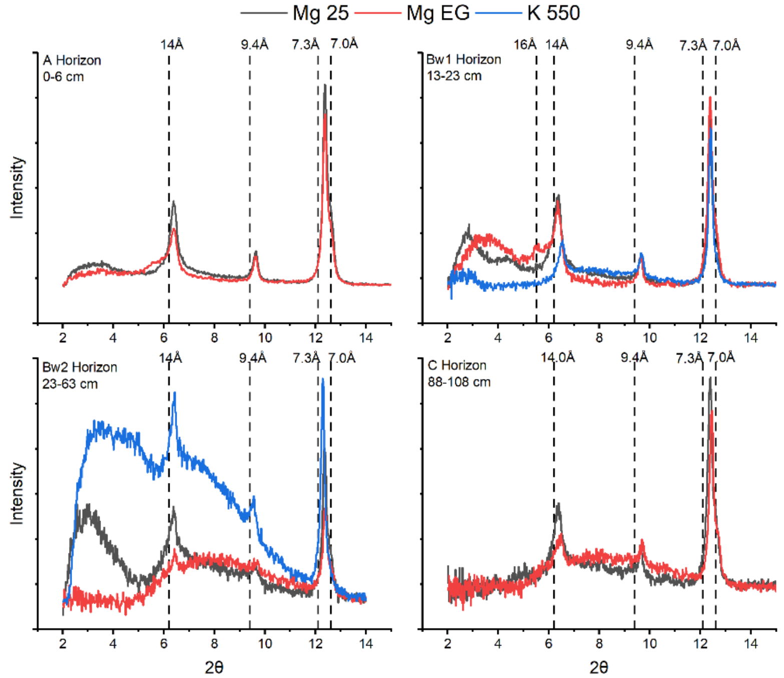

Figure S14 – Swift Creek Middle clay-size fraction oriented XRD patterns measured after treatment with Mg saturation followed by air-drying (MG 25), Mg saturation followed by Ethylene glycolation (Mg EG), and K saturation followed by heating at 550 °C (K550) as described in the online methods. Marked d-spacings correspond to phyllosilicate peak ID in table S2; 7.3 Å for serpentine, 14 Å and 7.0 Å for chlorite, 9.4 Å for talc. Minor shifts to ~16 Å following glycolation in the 13-23 cm sample are consistent with smectite presence.

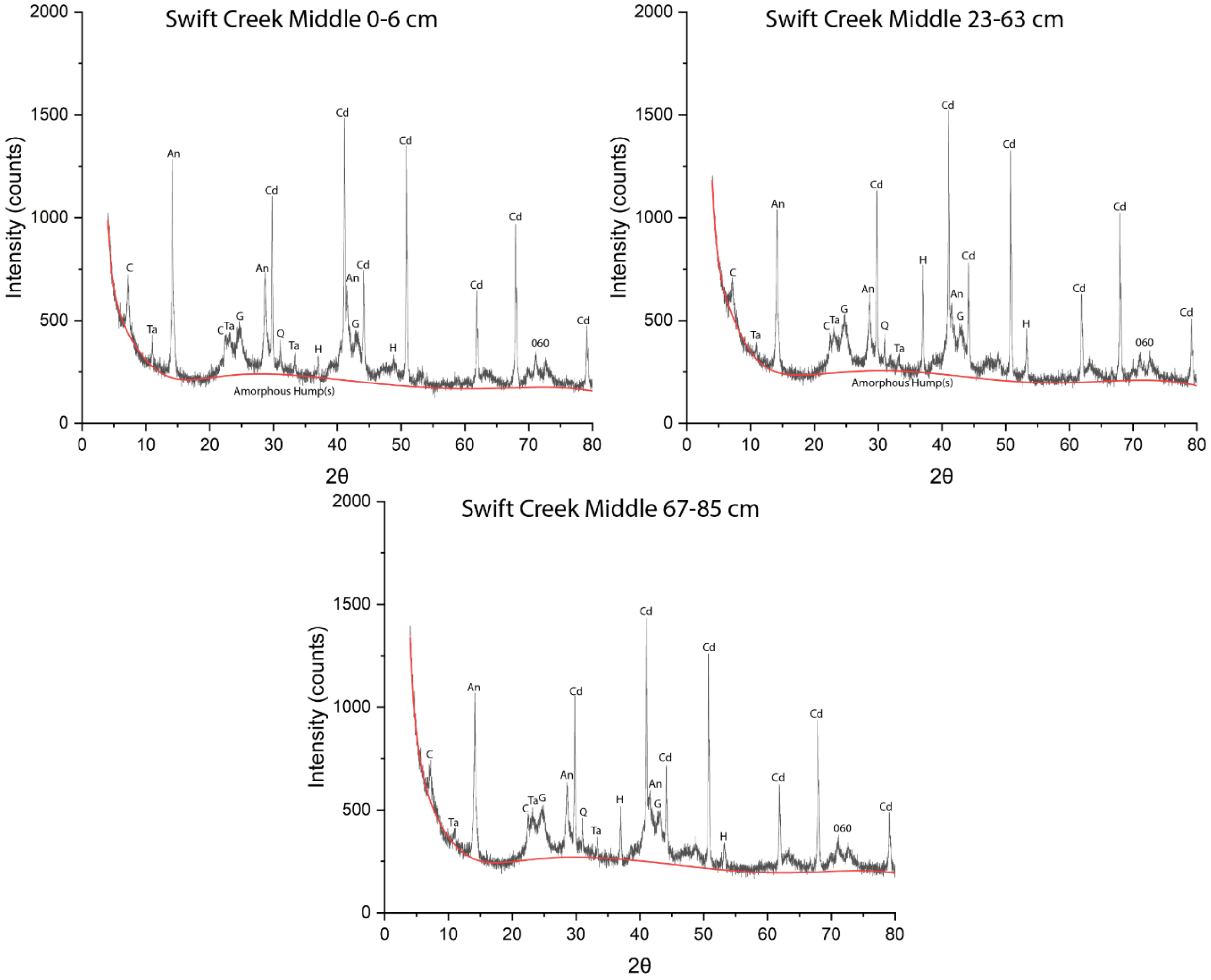

Figure S15: Labelled XRD patterns (Co Kα source) from randomly oriented mounts of soil clay-size fraction material from the Swift Creek Middle soil in the Klamath Mountains. The background plus amorphous determined by Rietveld refinement is given in red. The locations of the amorphous hump(s) are marked. Abbreviations: Antigorite (An), Chlorite (C), Corundum (Cd), Goethite (G), Halite (H), Quartz (Q), Talc (Ta). 060 peaks associated with phyllosilicates are marked as well. Corundum peaks in the clay-size fraction result from spiking for Rietveld refinement. Halite peaks represent minor residual presence from the clay-size extraction procedure. Smectites are not definitively observed in randomly oriented powder samples but are potentially present based on oriented clay mount observations (Figure S14).

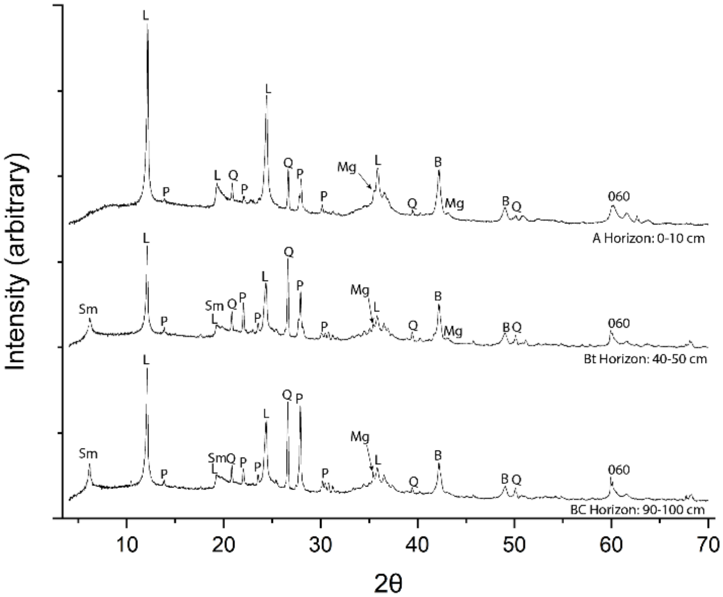

Figure S16 – String Bean Creek randomly oriented XRD patterns from mounts of bulk soil (left, Cu Kα source). Abbreviations: Brass (B), Lizardite (L), Magnetite, Quartz (Q), Plagioclase (P), and Smectites (Sm). 060 peaks associated with phyllosilicates are marked as well. Peaks marked B result from using a Brass sample stage for XRD analysis.

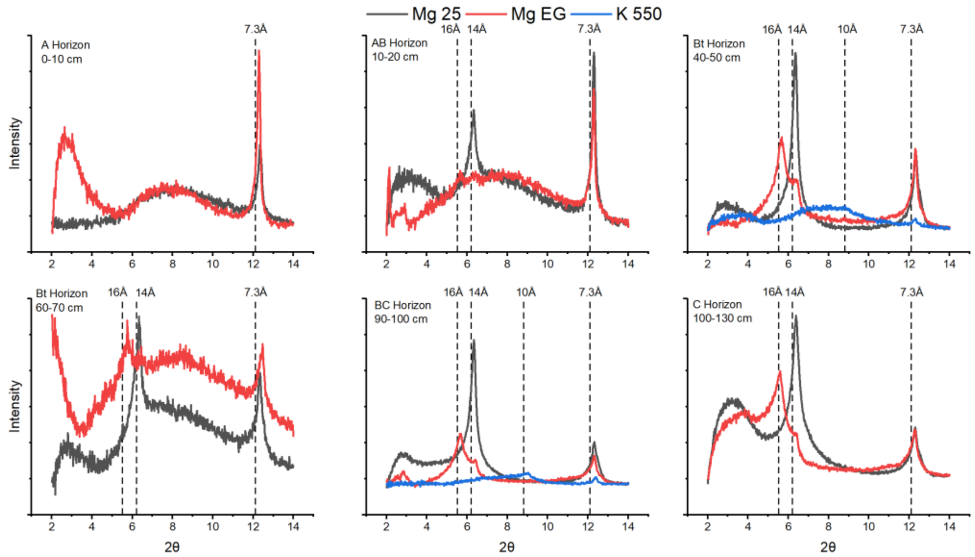

Figure S17 – String Bean Creek clay-size fraction oriented XRD patterns measured after treatment with Mg saturation followed by air-drying (MG 25), Mg saturation followed by Ethylene glycolation (Mg EG), and K saturation followed by heating at 550 °C (K550) as described in the online methods. Panels are top left) A horizon from 0-10 cm, top middle) AB horizon from 10-20 cm, top right) Bt horizon from 40-50 cm, Bottom left) Bt horizon from 60-70 cm, Bottom Middle) BC horizon from 90-100 cm, and Bottom Right) C horizon from 100-130 cm. These depths were chosen to include at least 1 depth from within each identified soil horizon. Mg 25 stands for magnesium saturated and air dried, Mg EG stands for magnesium saturated and ethylene glycol vapor solvated, and K 550 stands for K saturated and heated to 550℃. Dashed lines correspond to ideal d-spacings for phyllosilicates in Table S2; ~7.3 Å for serpentine, ~14 Å for air dried smectite, ~16 Å for glycolated smectite, ~10 Å for smectites heated to 550℃. Residual peaks at ~14 Å following ethylene glycol solvation are attributed to the presence of high charge smectites.

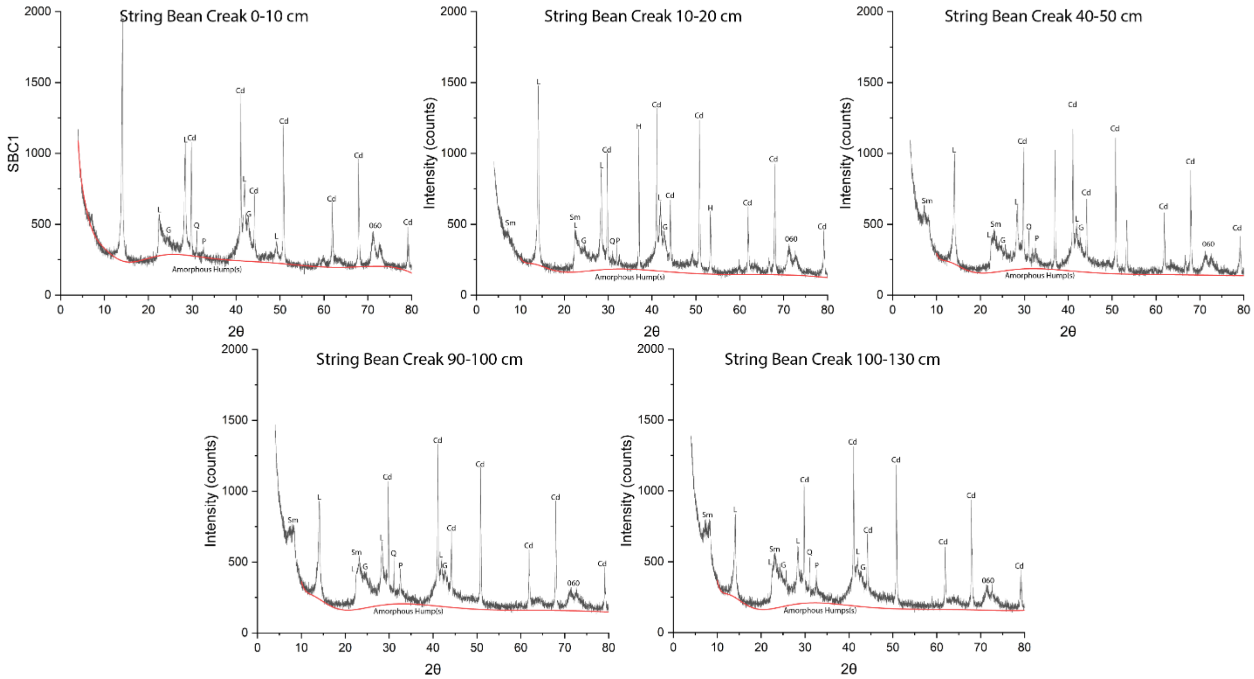

Figure S18: Labelled XRD patterns (Co Kα source) from randomly oriented mounts of soil clay-size fraction material from the String Bean Creek soil in the Klamath Mountains. The background plus amorphous determined by Rietveld refinement is given in red. The locations of the amorphous hump(s) are marked. Abbreviations: Corundum (Cd), Goethite (G), Halite (H), Quartz (Q), Plagioclase (P), Smectites (Sm). 060 peaks associated with phyllosilicates are marked as well. Corundum peaks in the clay-size fraction result from spiking for Rietveld refinement. Halite peaks represent minor residual presence from the clay-size extraction procedure.

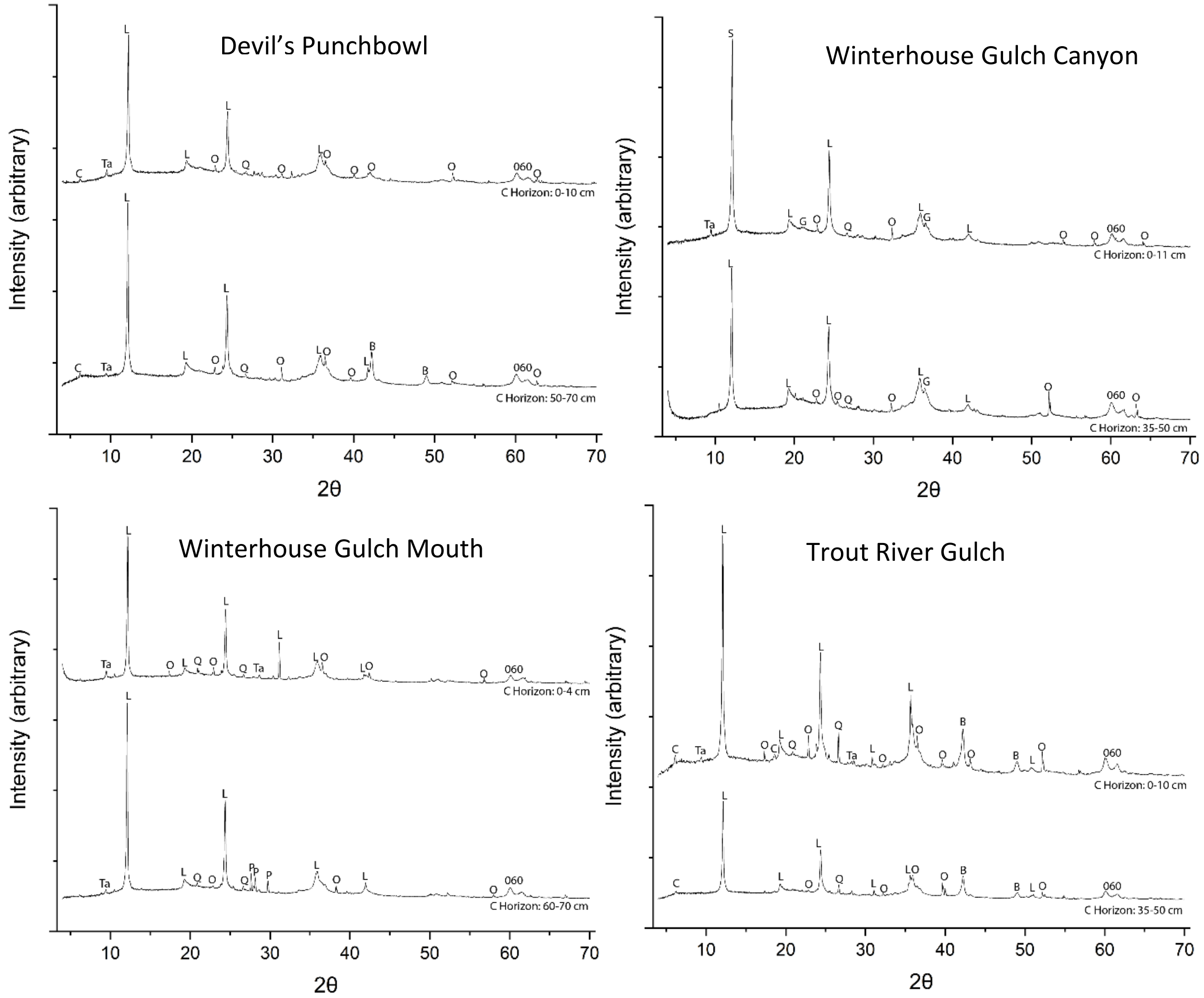

Figure S19 – XRD patterns from randomly oriented mounts of bulk soil material (Cu Kα source) from the Tablelands. Abbreviations: Brass (B), Chlorite (C), Lizardite (L), Olivine (O), Quartz (Q), and Talc (Ta). 060 peaks associated with phyllosilicates are marked as well. Peaks marked B result from using a Brass sample stage for XRD analysis.

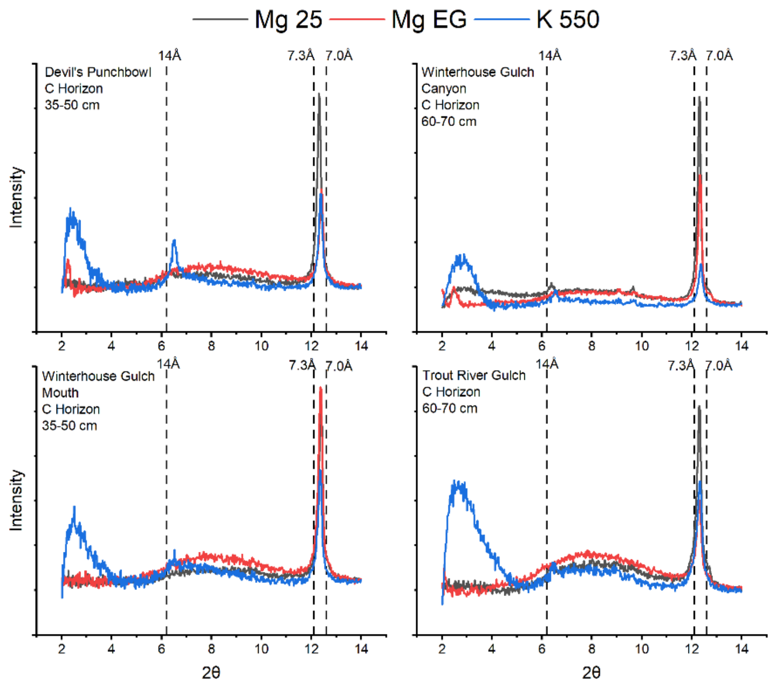

Figure S20 – Tablelands clay-size fraction oriented XRD patterns measured after treatment with Mg saturation followed by air-drying (MG 25), Mg saturation followed by Ethylene glycolation (Mg EG), and K saturation followed by heating at 550 °C (K550) as described in the online methods. Marked d-spacings correspond to phyllosilicate peak ID in Table S2, 7.3 Å for serpentine, 14 Å and 7.0Å for chlorite.

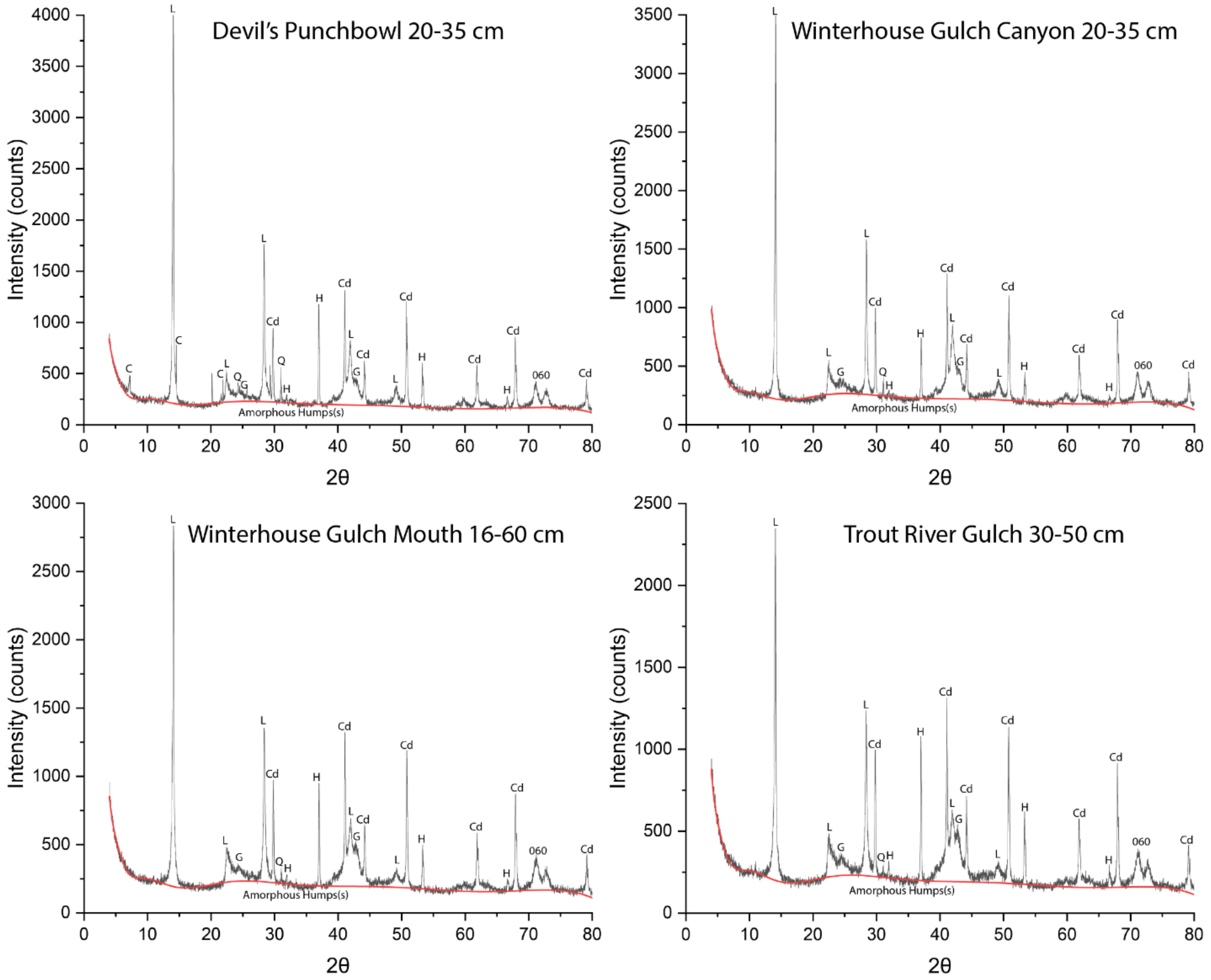

Figure S21: Labelled XRD patterns (Co Kα source) from randomly oriented mounts of soil clay-size fraction material from all soils in the Tablelands. The background plus amorphous determined by Rietveld refinement is given in red. The locations of the amorphous hump(s) are marked. Abbreviations: Corundum (Cd), Goethite (G), Halite (H), Lizardite (L), Quartz (Q). 060 peaks associated with phyllosilicates are marked as well. Corundum peaks in the clay-size fraction result from spiking for Rietveld refinement. Halite peaks represent minor residual presence from the clay-size extraction procedure.

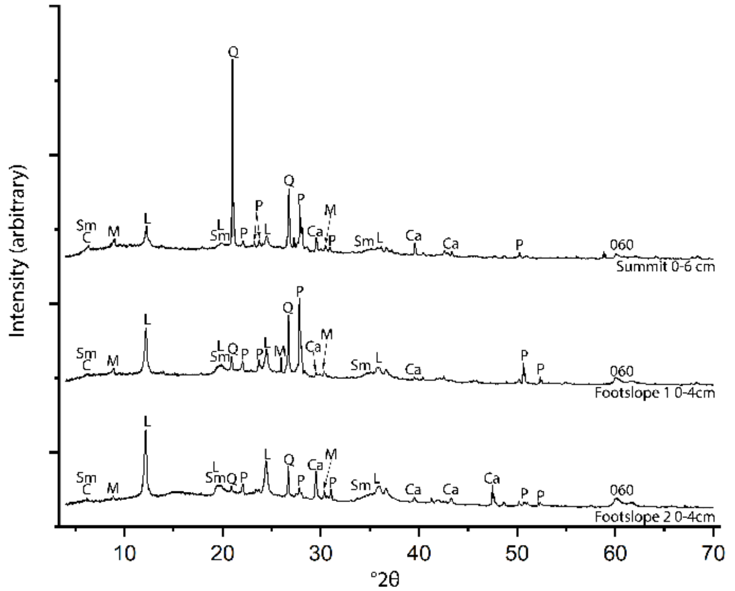

Figure S22 –Labelled XRD patterns (Cu Kα source) of randomly oriented mounts of the bulk soil from all soil pits at Pickhandle Gulch. Abbreviations: Calcite (Ca), Chlorite (C), Corundum (Cd), Lizardite (L), Muscovite (M), Plagioclase (P), Quartz (Q), Talc (Ta), and Smectites (Sm).

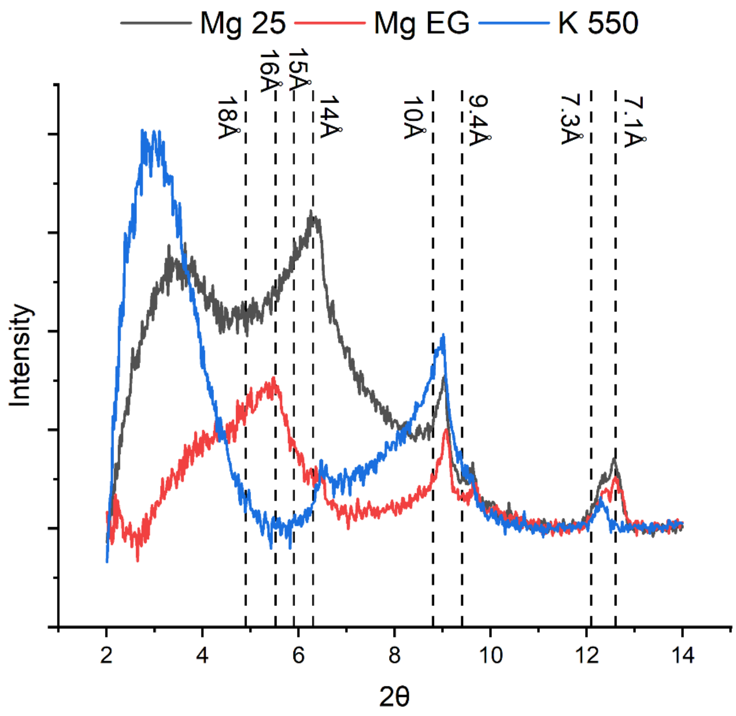

Figure S23 – Clay-size fraction oriented XRD patterns from the Pickhandle Gulch Footslope 1 soil C horizon (4-8 cm) after treatment with Mg saturation followed by air-drying (MG 25), Mg saturation followed by Ethylene glycolation (Mg EG), and K saturation followed by heating at 550 °C (K550) as described in the online methods. Dashed lines correspond to ideal d-spacings for phyllosilicate in Table S2; ~14 Å for chlorite (001) and ~7.0 Å for chlorite (002), ~7.3 Å for serpentine (001), ~9.4 Å for Talc (001), 10 Å Muscovite (001), ~15 Å for air dried smectite (001) and 16 Å for ethylene glycol solvated smectite (001) and 10 Å for smectite (001) heated to 550℃.

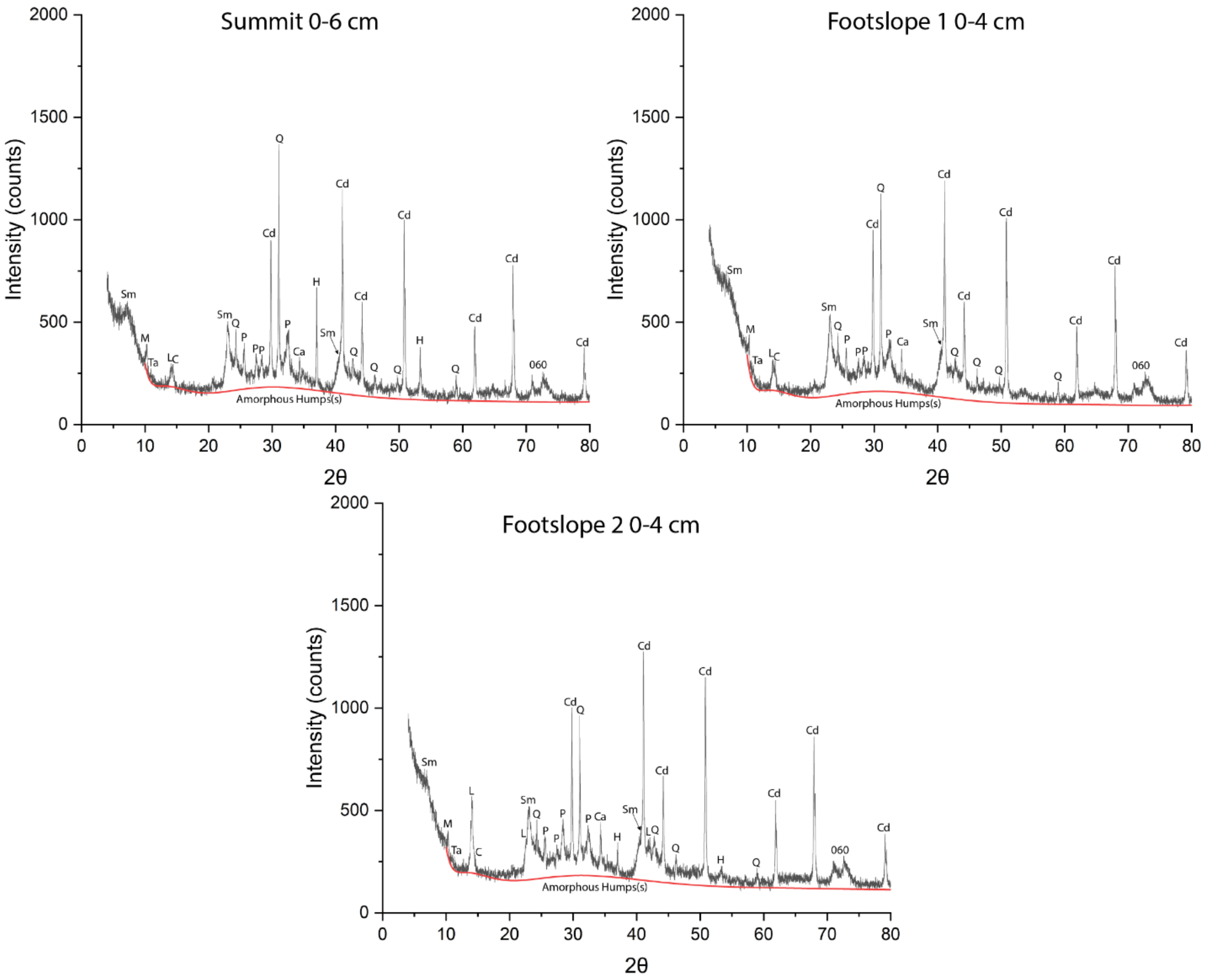

Figure S24: Labelled XRD patterns (Co Kα source) from randomly oriented mounts of soil clay-size fraction material from all soils at Pickhandle Gulch. The background plus amorphous determined by Rietveld refinement is given in red. The locations of the amorphous hump(s) are marked. Abbreviations: Chlorite (C), Corundum (Cd), Halite (H), Lizardite (L), Muscovite (M), Plagioclase (P), Quartz (Q), Smectites (Sm), Talc (Ta). 060 peaks associated with phyllosilicates are marked as well. Corundum peaks in the clay-size fraction result from spiking for Rietveld refinement. Halite peaks represent minor residual presence from the clay-size extraction procedure.

S1.5 Rietveld Refinements of 20 wt.% α-Al_2O_3 spiked Clay-Size Fraction Samples

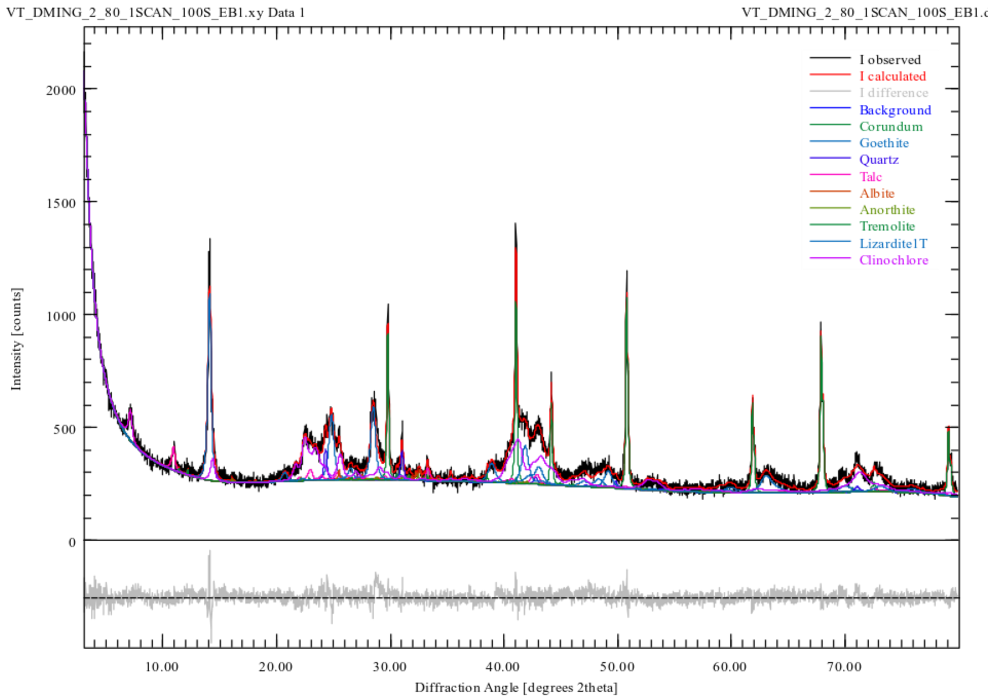

Figure S25 – Rietveld refinement of the randomly oriented clay-size fraction from 0-3 cm depth in the Eunice Bluff soil in the Klamath Mountains spiked with 20% α-Al_2O_3. Pattern measured using a Co Kα source.

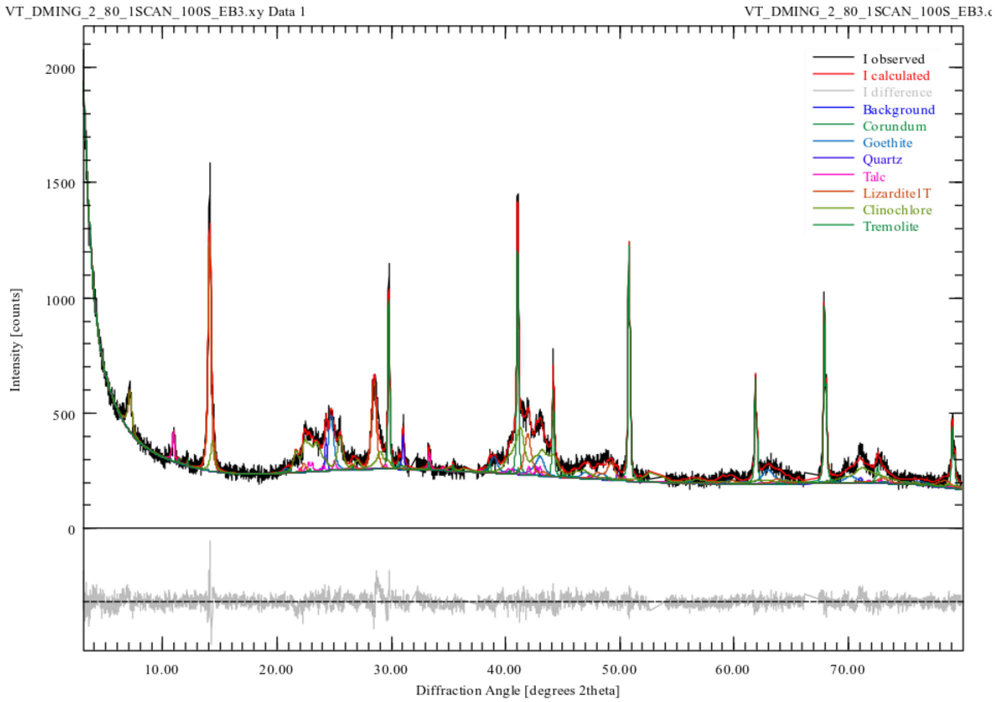

Figure S26 – Rietveld refinement of the randomly oriented clay-size fraction from 13-23 cm depth in the Eunice Bluff soil in the Klamath Mountains spiked with 20% α-Al_2O_3. Pattern measured using a Co Kα source.

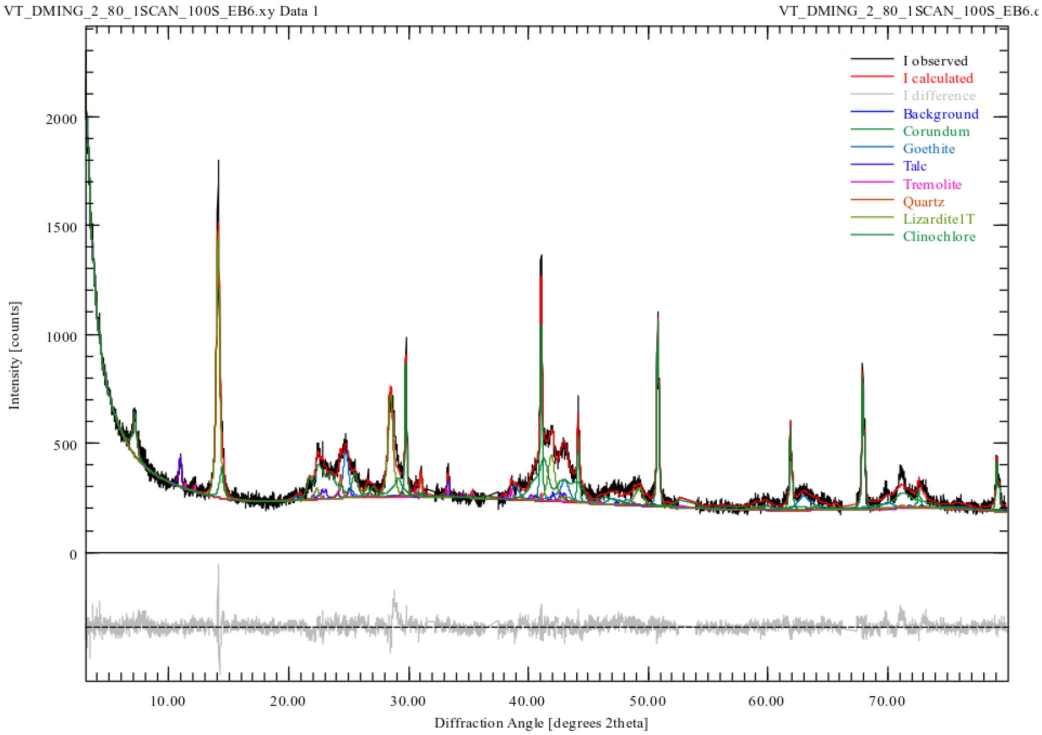

Figure S27 – Rietveld refinement of the randomly oriented clay-size fraction from 43-63 cm depth in the Eunice Bluff soil in the Klamath Mountains spiked with 20% α-Al_2O_3. Pattern measured using a Co Kα source.

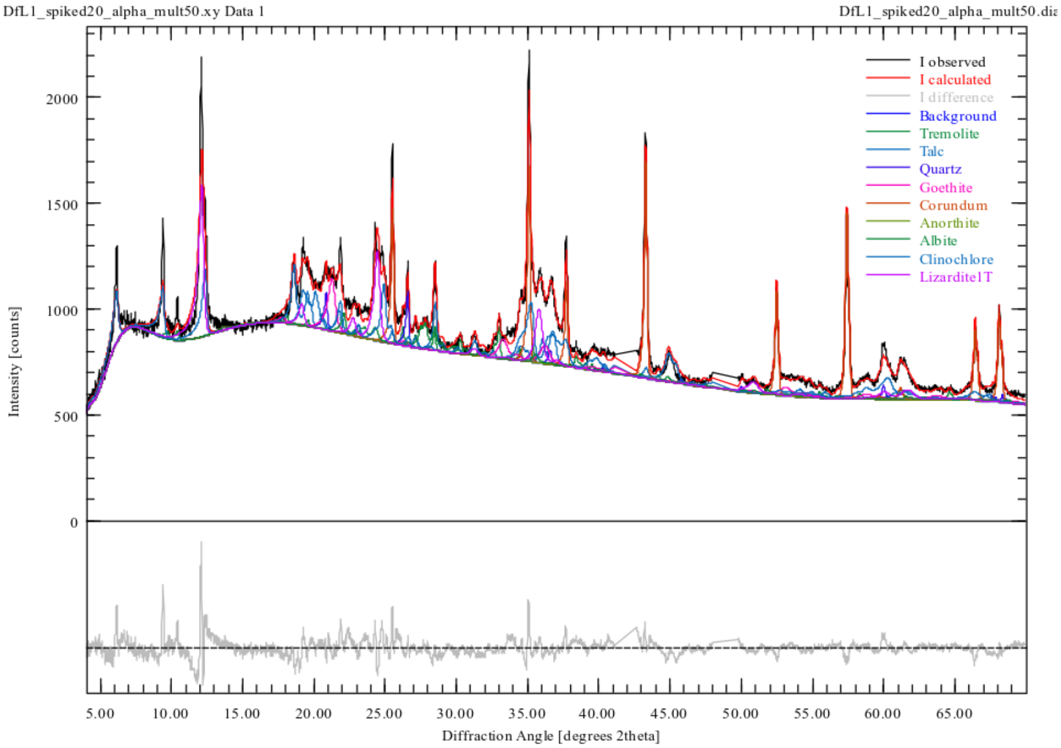

Figure S28 – Rietveld refinement of the randomly oriented clay-size fraction from 0-7 cm depth in the Deadfall Lake soil in the Klamath Mountains spiked with 20% α-Al_2O_3. Pattern measured using a Cu Kα source between 4 and 70 $^o2\theta$. Pattern intensity multiplied by 50x prior to refinement fitting and fit between 4 and 70 $^o2\theta$.

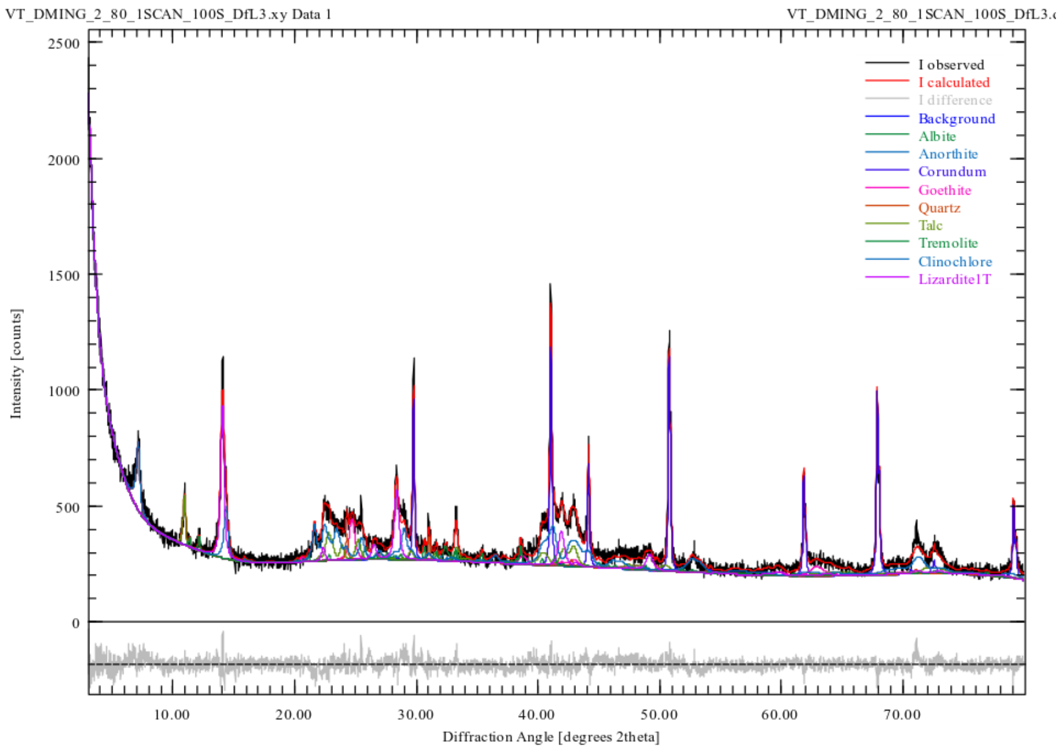

Figure S29 – Rietveld refinement of the randomly oriented clay-size fraction from 17-27 cm depth in the Deadfall Lake soil in the Klamath Mountains spiked with 20% α-Al_2O_3. Pattern measured using a Co Kα source.

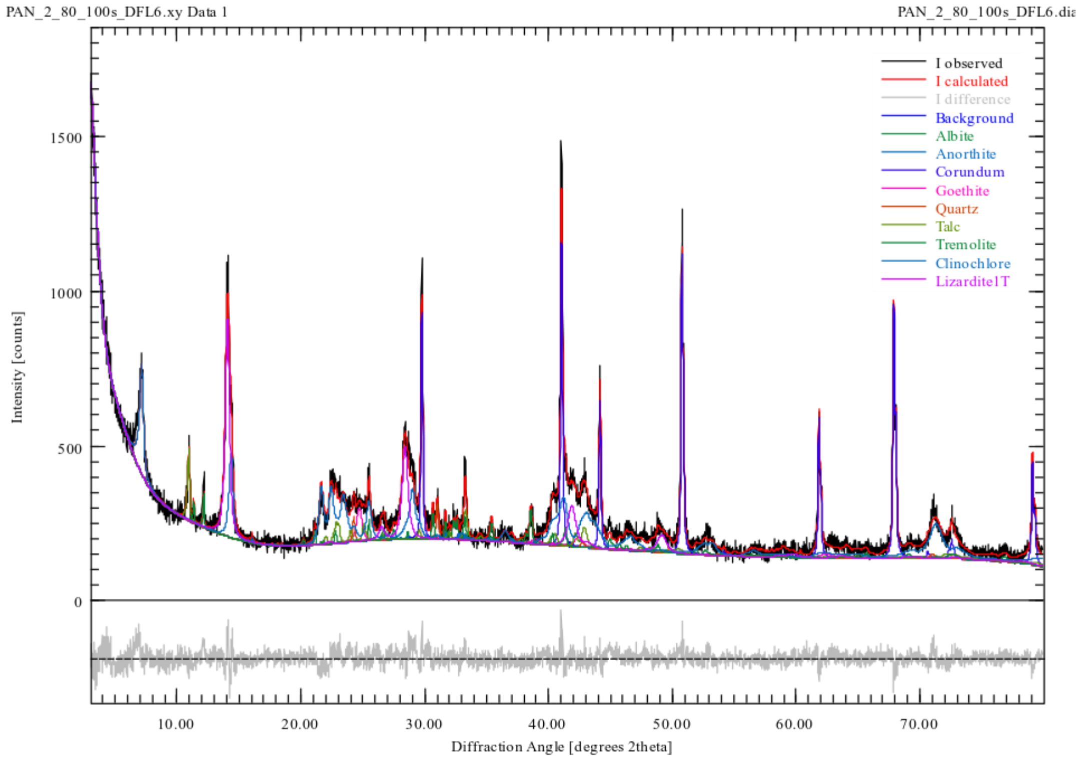

Figure S30 – Rietveld refinement of the randomly oriented clay-size fraction from 47-53 cm depth in the Deadfall Lake soil in the Klamath Mountains spiked with 20% α-Al_2O_3. Pattern measured using a Co Kα source.

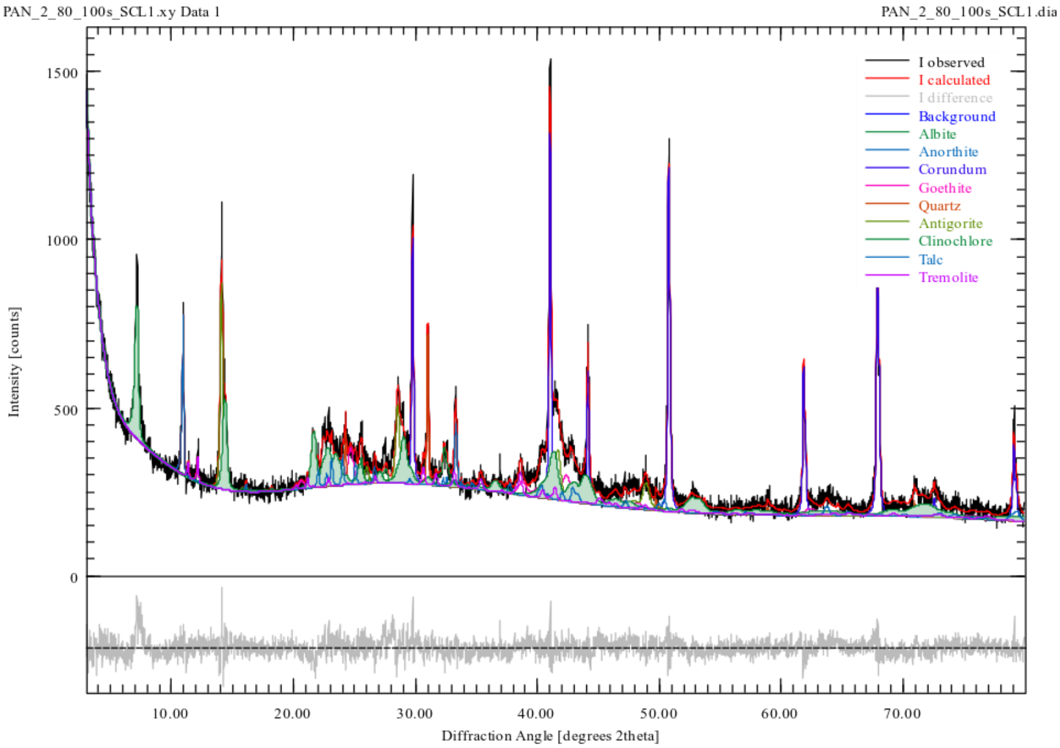

Figure S31 – Rietveld refinement of the randomly oriented clay-size fraction from 0-5 cm depth in the Swift Creek late soil in the Klamath Mountains spiked with 20% α-Al_2O_3. Pattern measured using a Co Kα source.

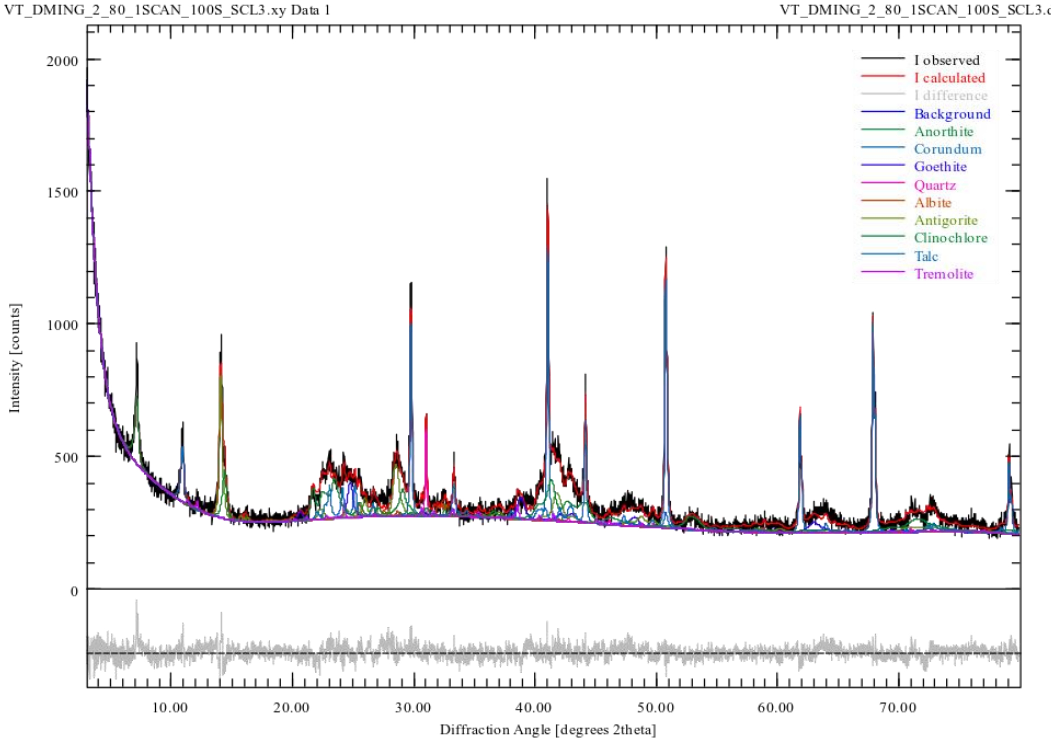

Figure S32 – Rietveld refinement of the randomly oriented clay-size fraction from 16-33 cm depth in the Swift Creek Late soil in the Klamath Mountains spiked with 20% α-Al_2O_3. Pattern measured using a Co Kα source.

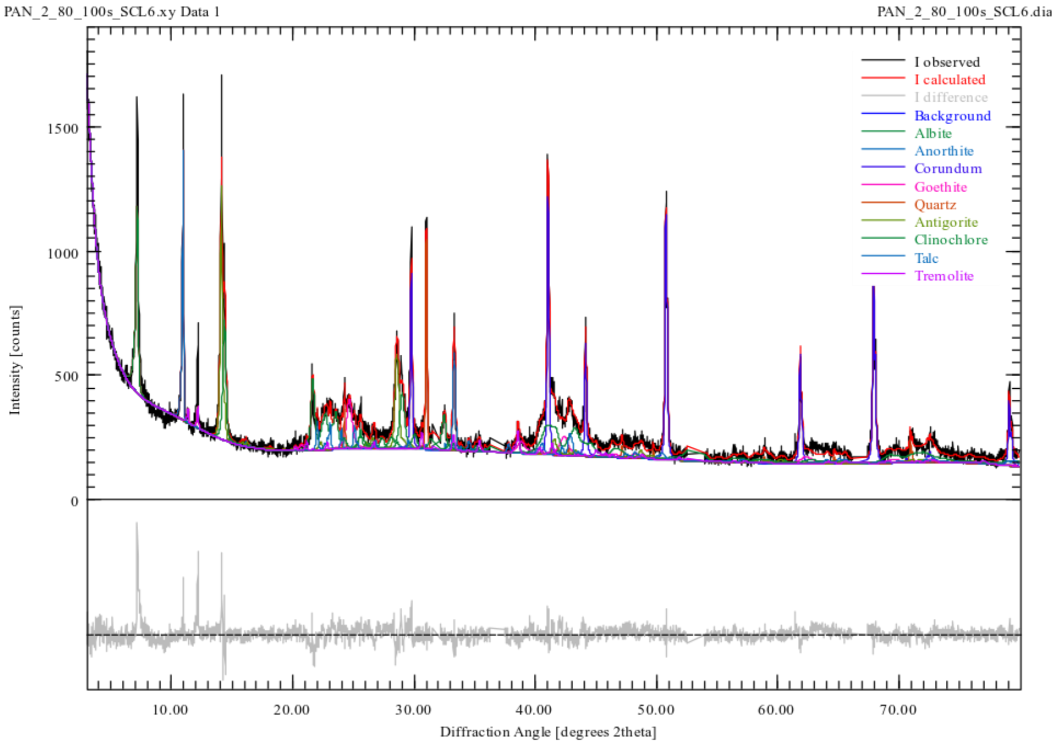

Figure S33 – Rietveld refinement of the randomly oriented clay-size fraction from 67-85 cm depth in the Swift Creek Late soil in the Klamath Mountains spiked with 20% α-Al_2O_3. Pattern measured using a Co Kα source.

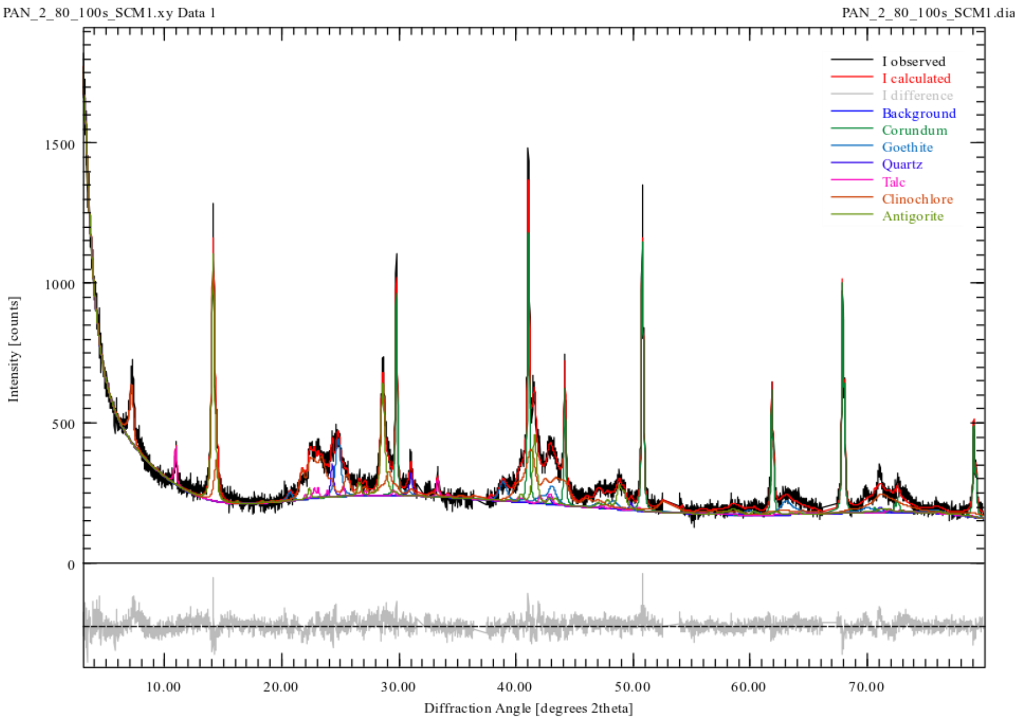

Figure S34 – Rietveld refinement of the randomly oriented clay-size fraction from 0-6 cm depth in the Swift Creek Middle soil in the Klamath Mountains spiked with 20% α-Al_2O_3. Pattern measured using a Co Kα source.

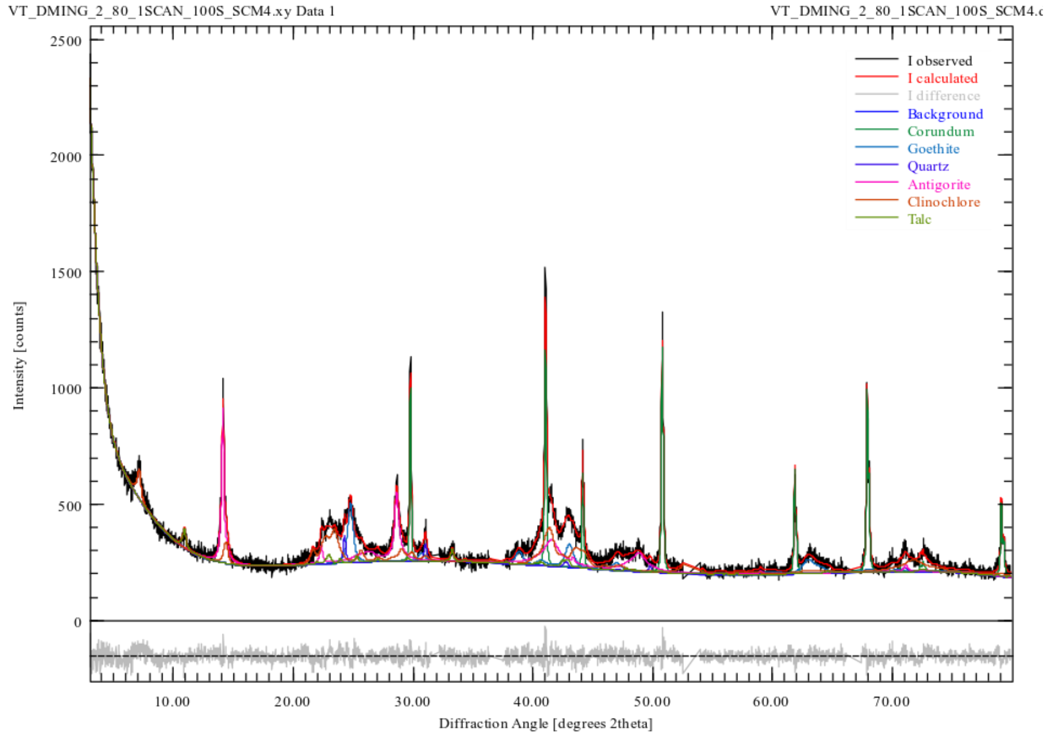

Figure S35 – Rietveld refinement of the randomly oriented clay-size fraction from 23-63 cm depth in the Swift Creek Middle soil in the Klamath Mountains spiked with 20% α-Al_2O_3. Pattern measured using a Co Kα source.

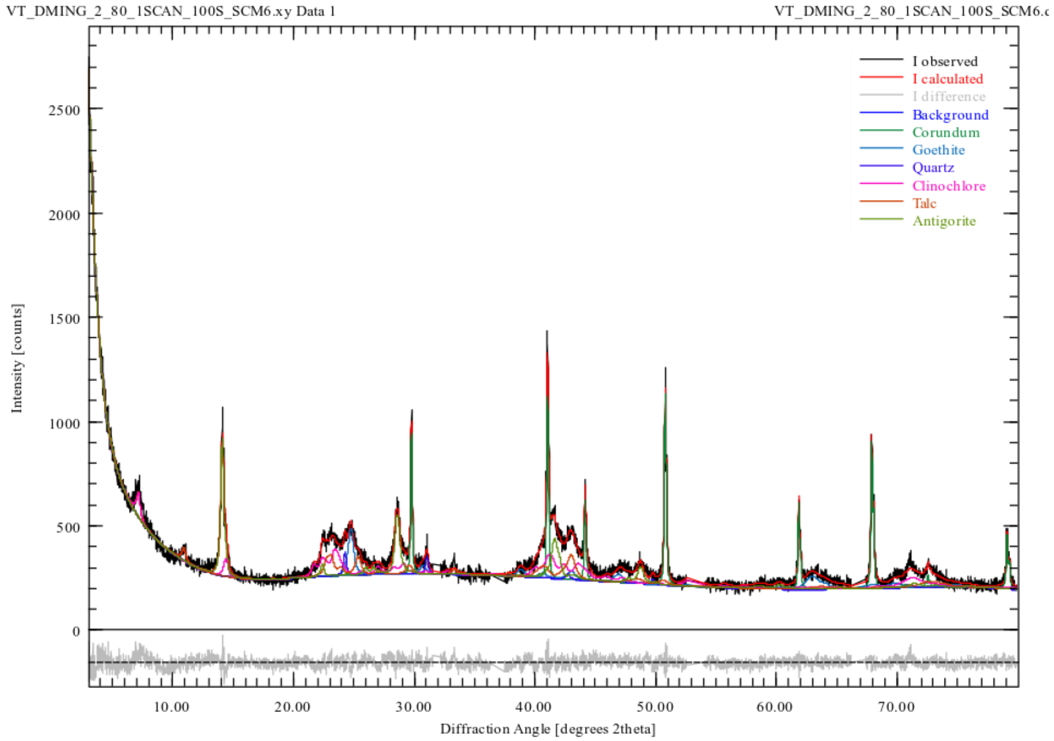

Figure S36 – Rietveld refinement of the randomly oriented clay-size fraction from 88-108 cm depth in the Swift Creek Middle soil in the Klamath Mountains spiked with 20% α-Al_2O_3. Pattern measured using a Co Kα source.

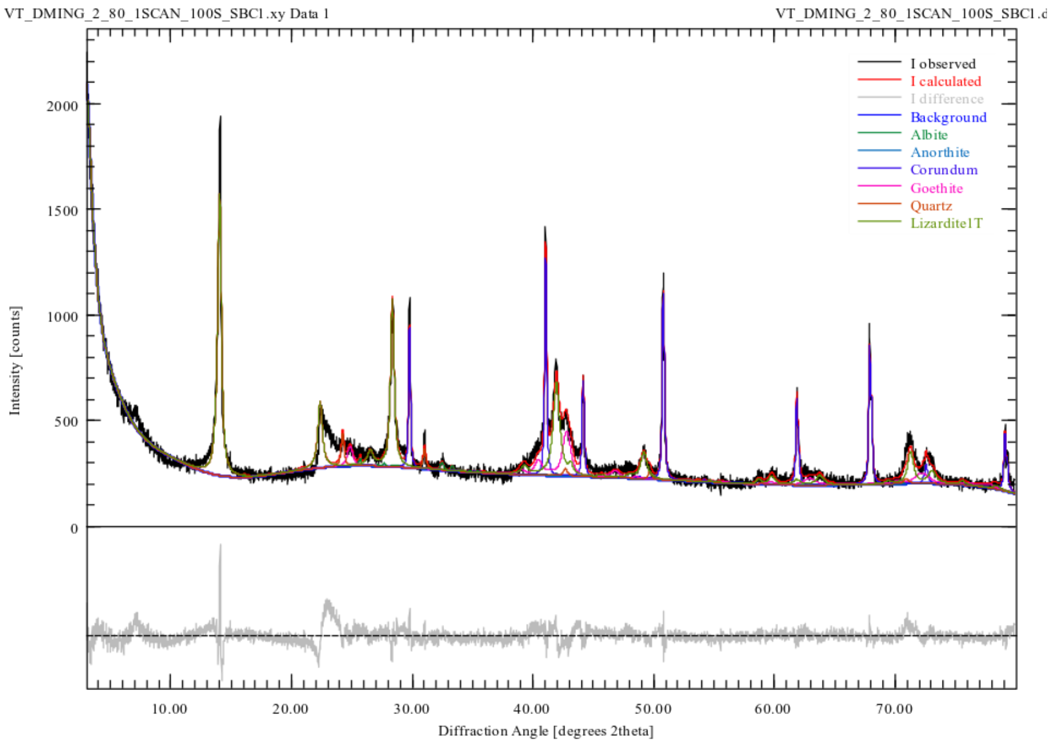

Figure S37 – Rietveld refinement of the randomly oriented clay-size fraction from 0-10 cm depth in the String Bean Creek soil in the Klamath Mountains spiked with 20% α-Al_2O_3. Pattern measured using a Co Kα source.

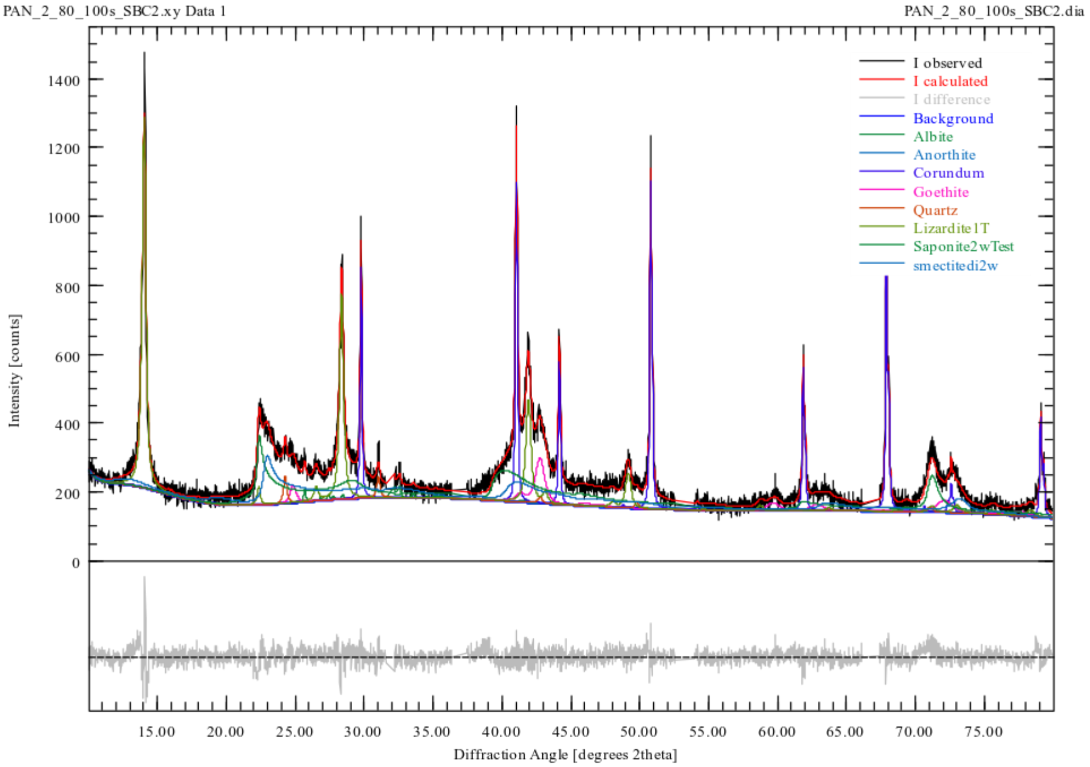

Figure S38 – Rietveld refinement of the randomly oriented clay-size fraction from 10-20 cm depth in the String Bean Creek soil in the Klamath Mountains spiked with 20% α-Al_2O_3. Pattern measured using a Co Kα source.

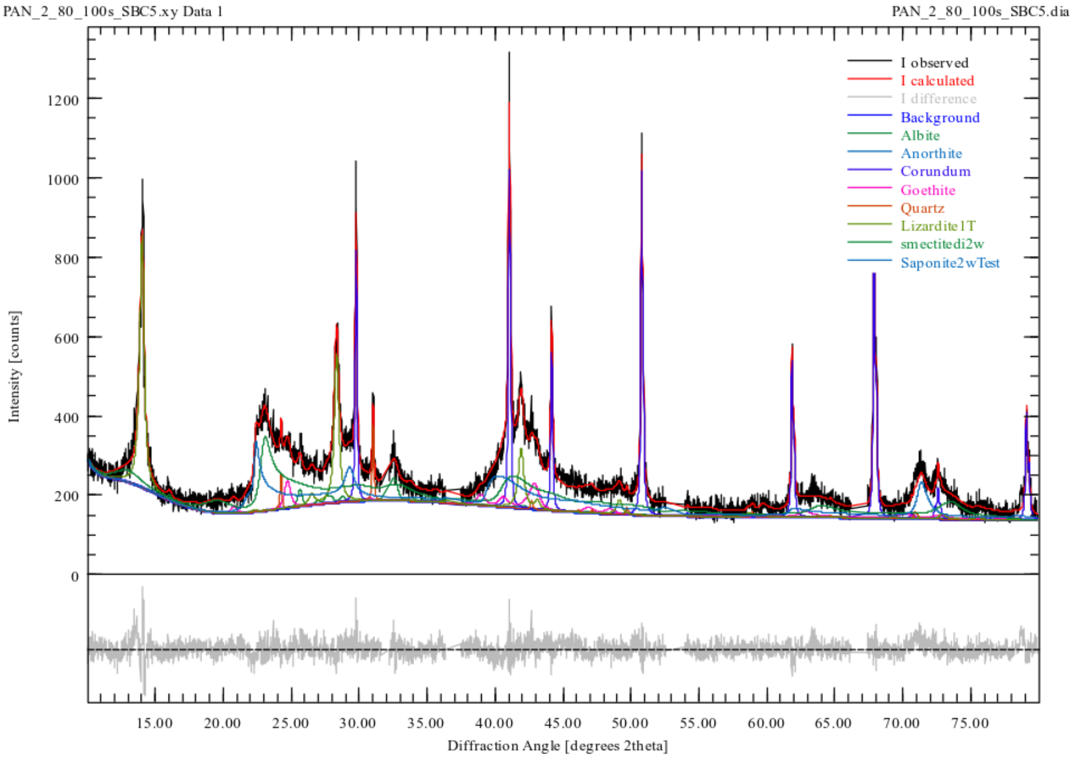

Figure S39 – Rietveld refinement of the randomly oriented clay-size fraction from 40-50 cm depth in the String Bean Creek soil in the Klamath Mountains spiked with 20% α-Al_2O_3. Pattern measured using a Co Kα source.

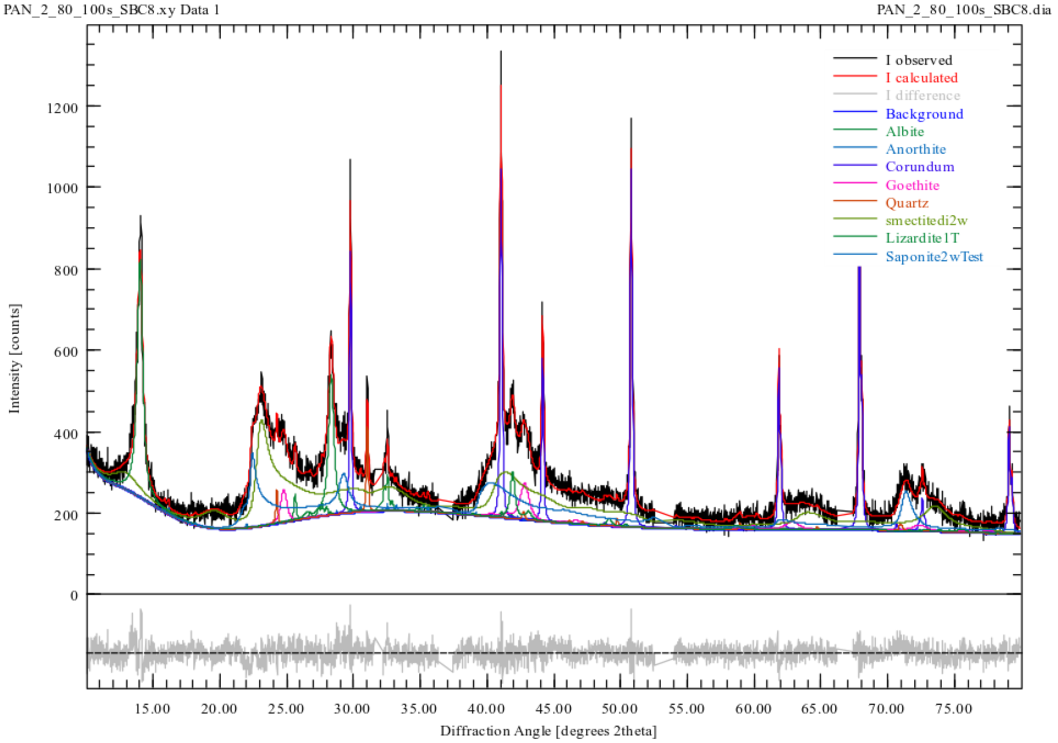

Figure S40 – Rietveld refinement of the randomly oriented clay-size fraction from 70-80 cm depth in the String Bean Creek soil in the Klamath Mountains spiked with 20% α-Al_2O_3. Pattern measured using a Co Kα source.

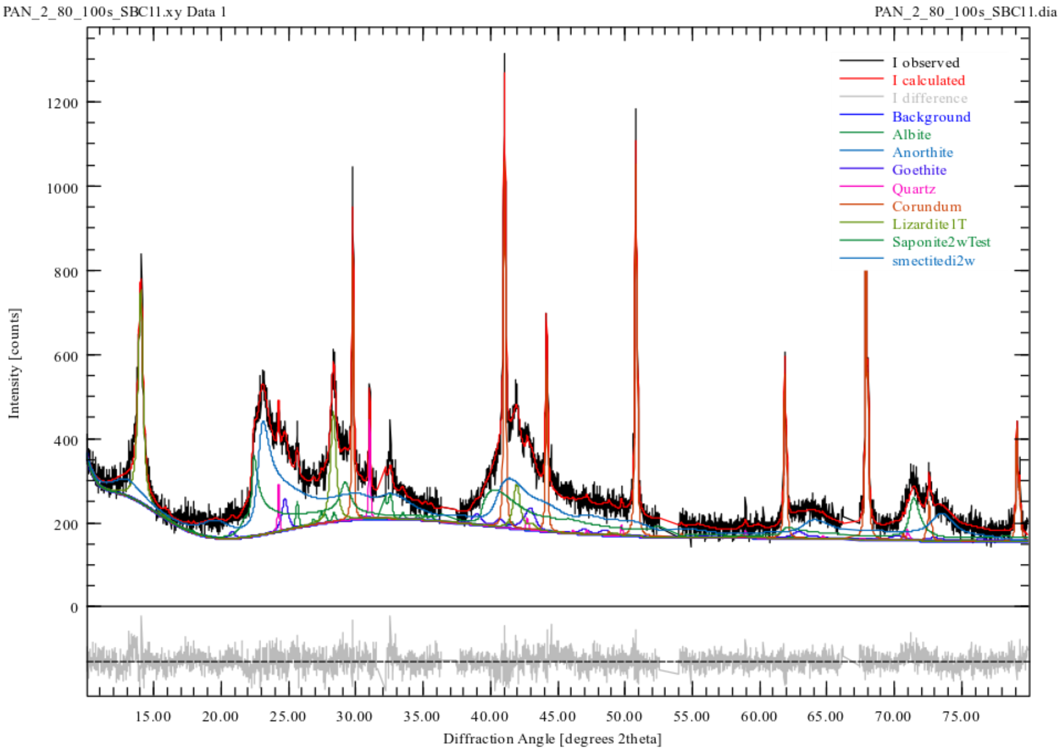

Figure S41 – Rietveld refinement of the randomly oriented clay-size fraction from 100-130 cm depth in the String Bean Creek soil in the Klamath Mountains spiked with 20% α-Al_2O_3. Pattern measured using a Co Kα source.

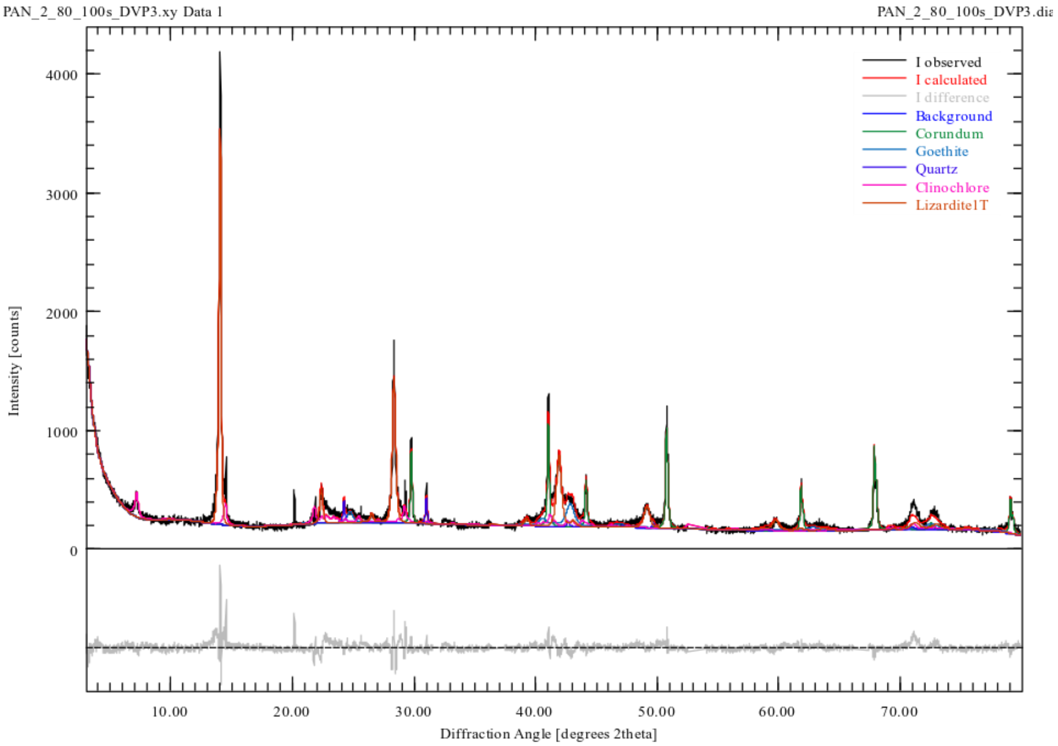

Figure S42 – Rietveld refinement of the randomly oriented clay-size fraction from 20-35 cm depth in the Devil's Punchbowl soil in the Tablelands spiked with 20% α-Al_2O_3. Pattern measured using a Co Kα source.

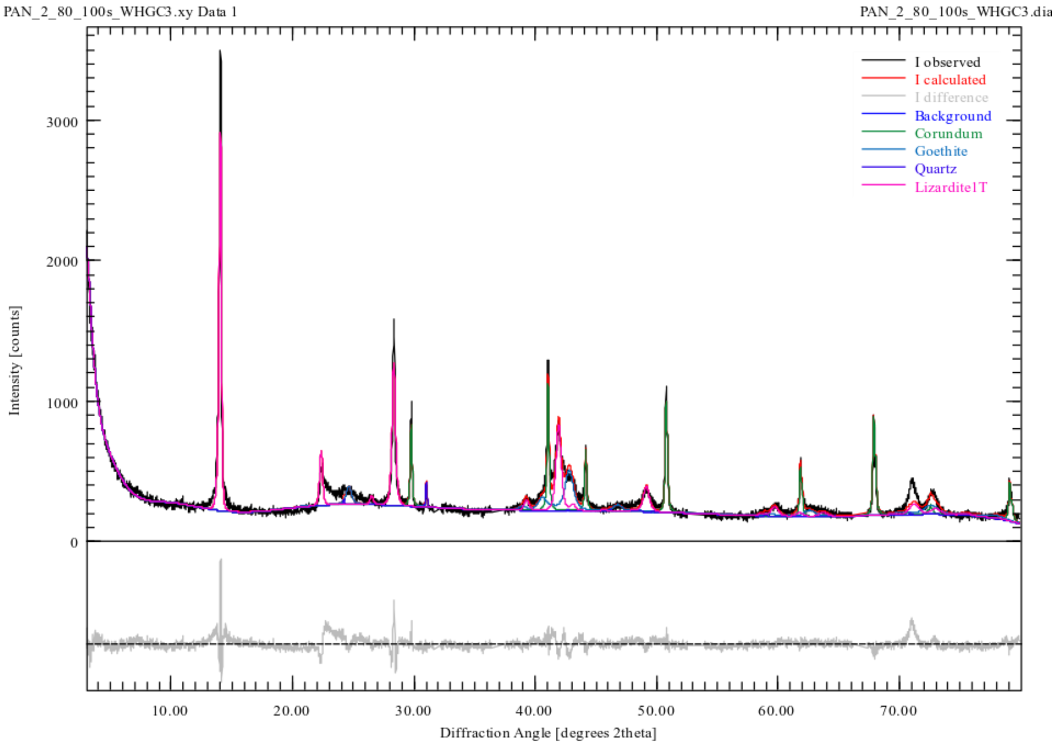

Figure S43 – Rietveld refinement of the randomly oriented clay-size fraction from 16-60 cm depth in the Winterhouse Gulch Canyon soil in the Tablelands spiked with 20% α-Al_2O_3. Pattern measured using a Co Kα source.

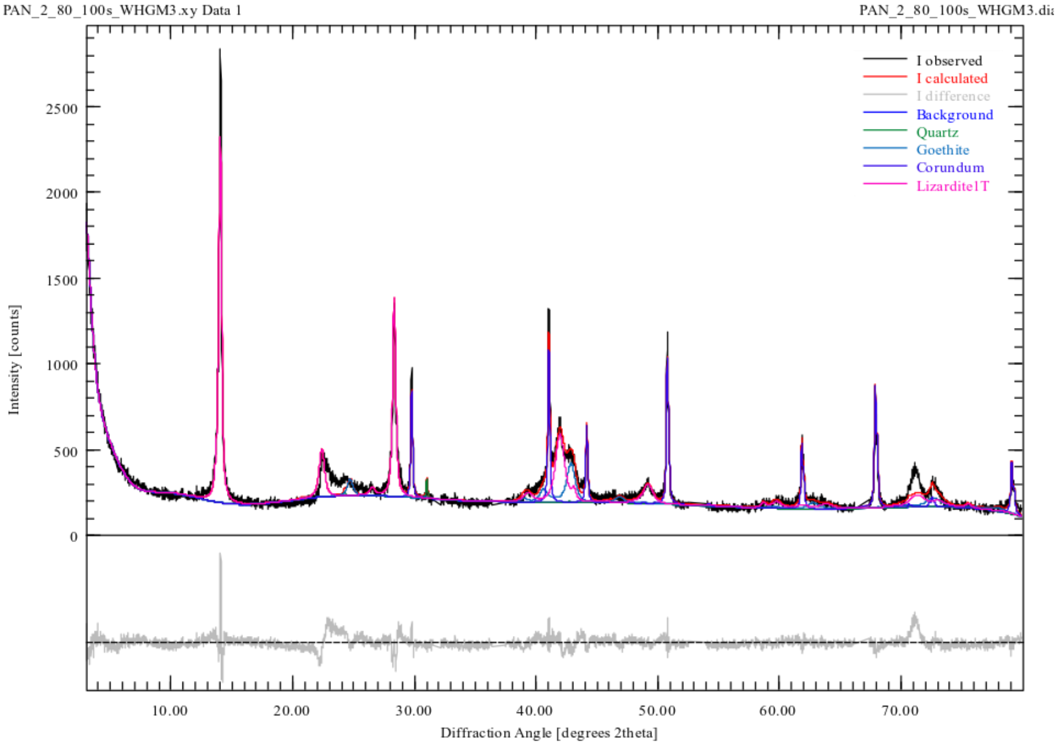

Figure S44 – Rietveld refinement of the randomly oriented clay-size fraction from 20-35 cm depth in the Winterhouse Gulch Mouth soil in the Tablelands spiked with 20% α-Al_2O_3. Pattern measured using a Co Kα source.

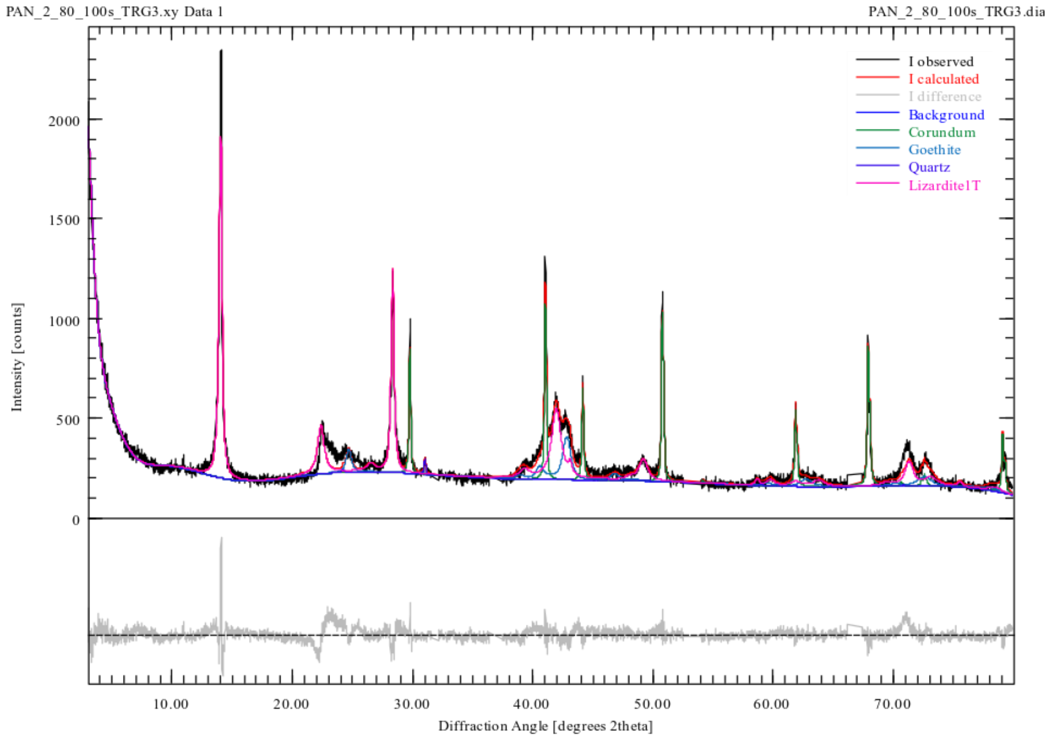

Figure S45 – Rietveld refinement of the randomly oriented clay-size fraction from 30-50 cm depth in the Trout River Gulch soil in the Tablelands spiked with 20% α-Al_2O_3. Pattern measured using a Co Kα source.

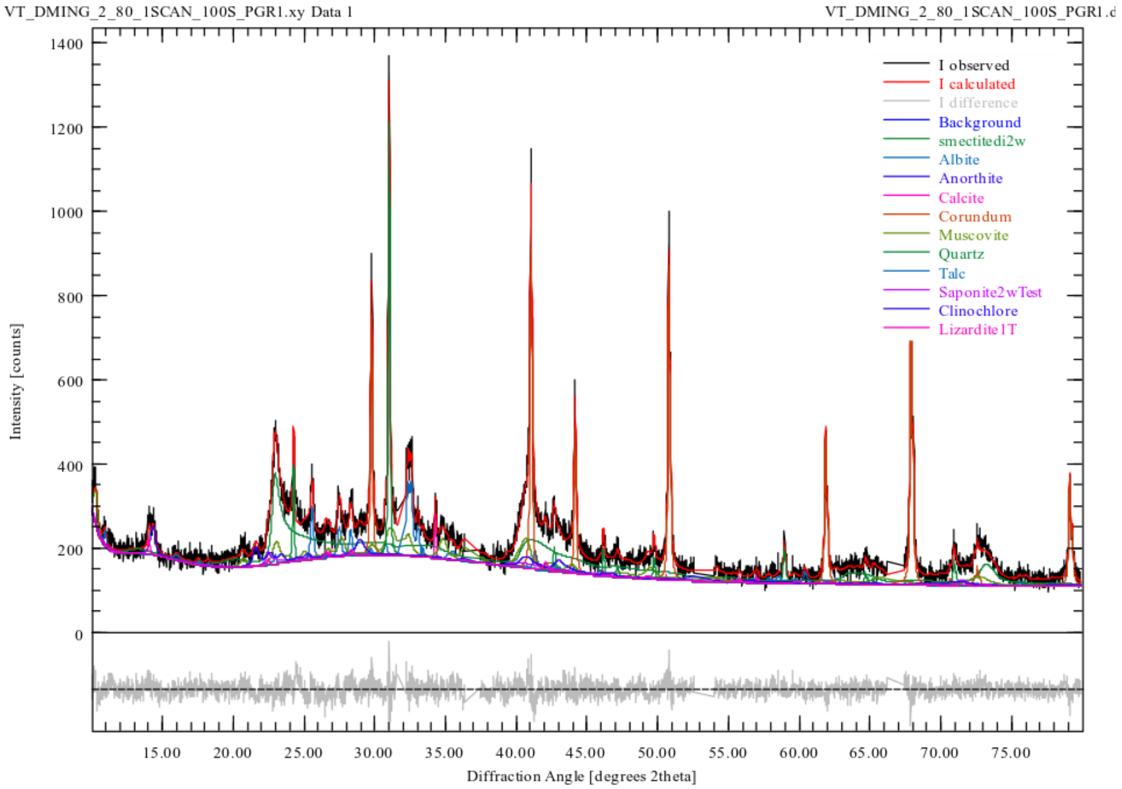

Figure S46 – Rietveld refinement of the randomly oriented clay-size fraction from 0-6 cm depth in the Summit soil at Pickhandle Gulch spiked with 20% α-Al_2O_3. Pattern measured using a Co Kα source.

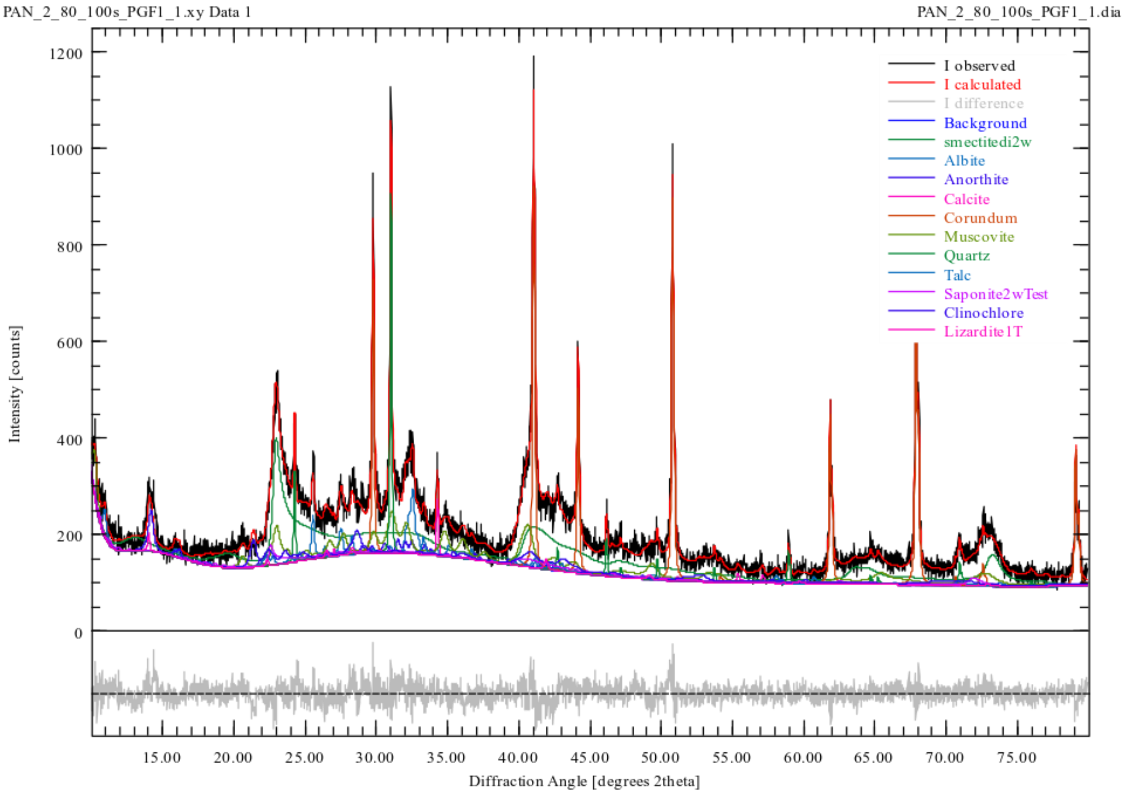

Figure S47 – Rietveld refinement of the randomly oriented clay-size fraction from 0-4 cm depth in the Footslope 1 soil at Pickhandle Gulch spiked with 20% α-Al_2O_3. Pattern measured using a Co Kα source.

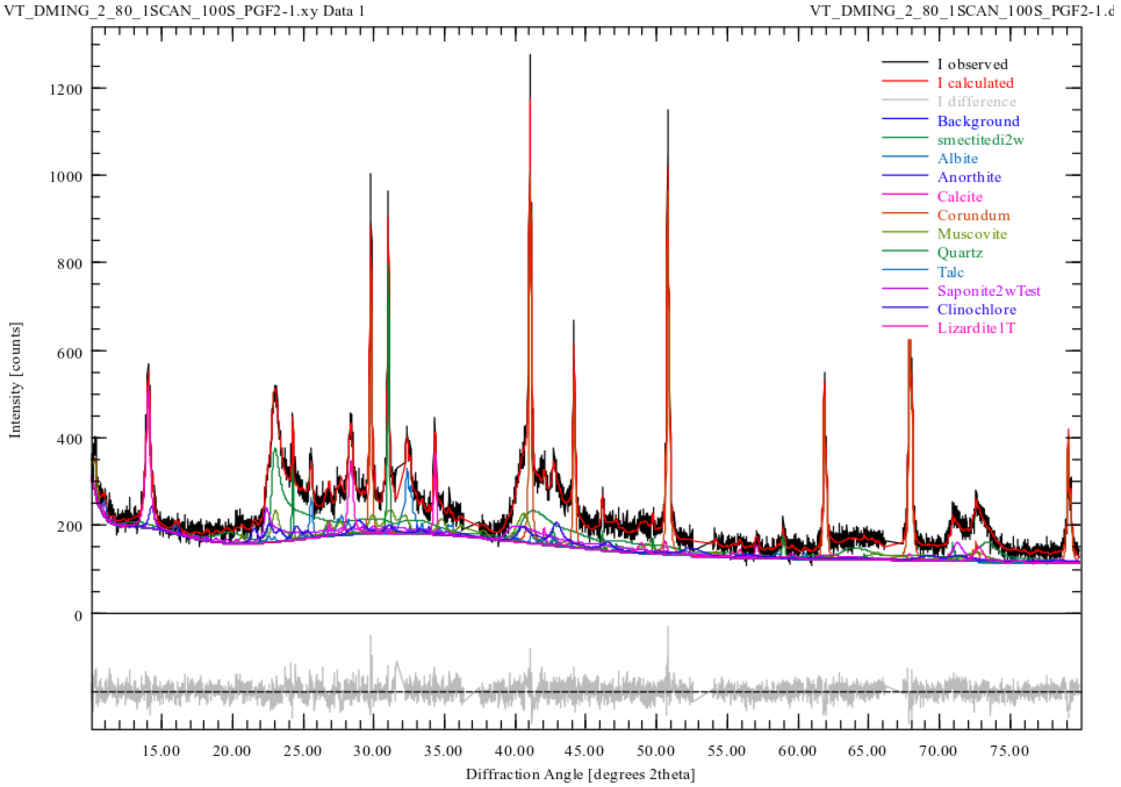

Figure S48 – Rietveld refinement of the randomly oriented clay-size fraction from 0-4 cm depth in the Footslope 2 soil at Pickhandle Gulch spiked with 20% α-Al_2O_3. Pattern measured using a Co Kα source.

S1.6 Additional Transmission Electron Microscopy Results

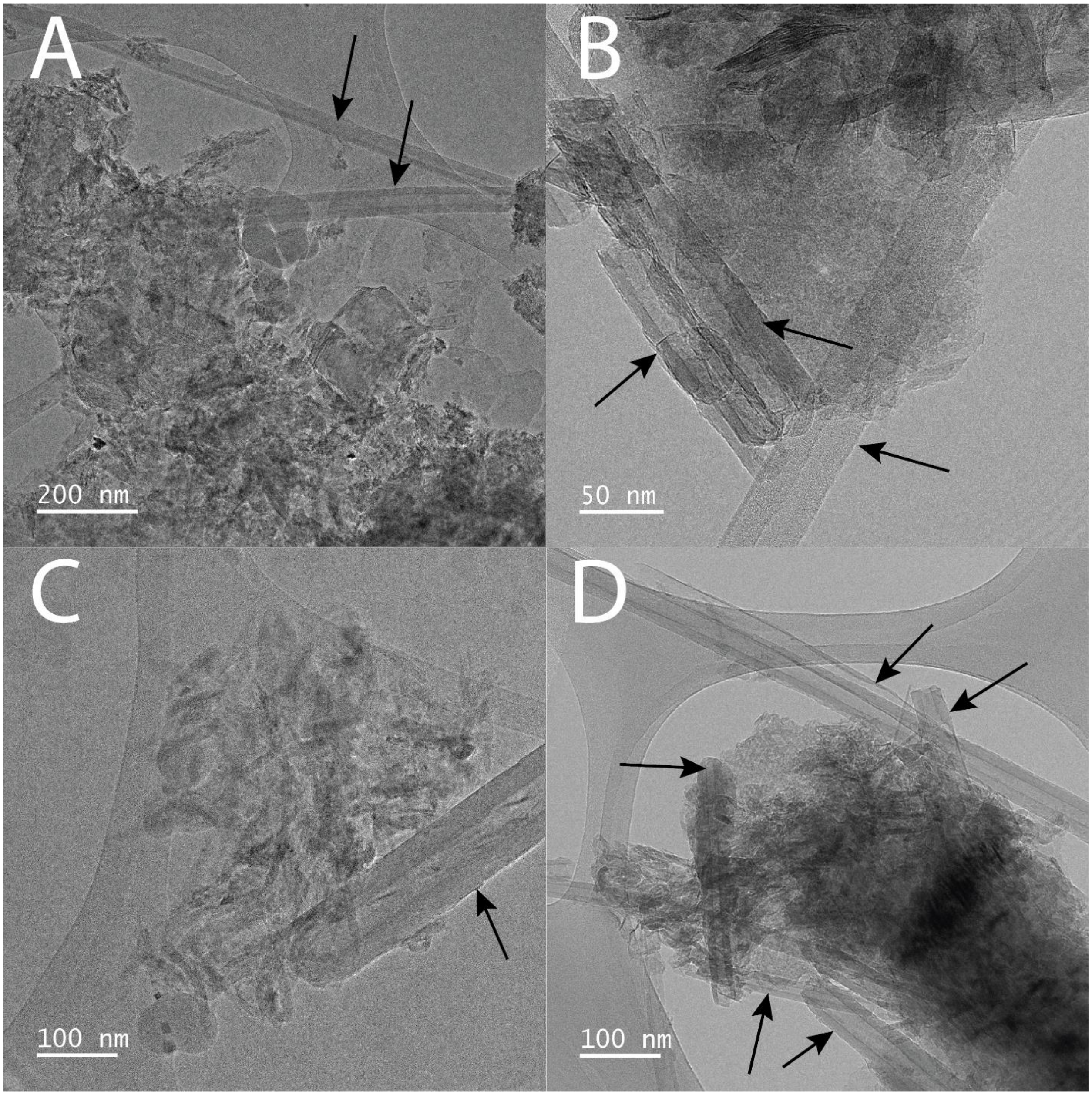

Figure S49 – TEM imagery of indicating the presence of fibrous chrysotile lathes in the clay-size fraction from the Eunice Bluff (A) and String Bean Creek (B) soils in the Klamath Mountains and the Devil's Punchbowl (C) and Trout River Gulch (D) soils in the Tablelands. Individual chrysotile fibers are marked by black arrows.

TEM demonstrates the presence of Fe-rich X-ray amorphous material in clay-size fraction material from both the Klamath Mountains and Tablelands (Figure 2 in main text). While the presence of serpentine minerals was confirmed through XRD (Figures S3-S24), chrysotile presence specifically is confirmed by TEM images demonstrating the presence of tubular/fibrous crystalline particles in the clay-size fraction of the Eunice Bluff and String Bean Creek soils in the Klamath Mountains and the Devil's Punchbowl and Trout River Gulch soils in the Tablelands (Figure S49).

S1.7 Hydroxylamine Hydrochloride Extractable Si

Hydroxylamine hydrochloride preferentially attacks Fe-containing amorphous material but also extracts Si associated with the Fe[21–23]. Amorphous Si content is elevated relative to parent material concentrations in all soils except for the Swift Creek soils in the Klamath Mountains (Figure S50). Amorphous Si content in the parent material at the Swift Creek sites is >5x mean Si_H in each Swift Creek soil, indicating significant loss of Si within amorphous material as parent material weathers. At the Deadfall Lake soil, mean Si_H is ~9.0x the parent material concentration, indicating significant accumulation of Si-containing amorphous material. Amorphous Si increases are more modest at other Klamath Mountain sites; ~1.7x at Eunice Bluff and ~3.1x at String Bean Creek. Soil Si_H relative to parent material is ~3.8x in the Tablelands and ~4.9x at Pickhandle Gulch, indicating increasing amorphous Si content as soils develop. Overall hydroxylamine extractable Si is much more similar between field sites than hydroxylamine extractable Fe (Figure 4 in main text).

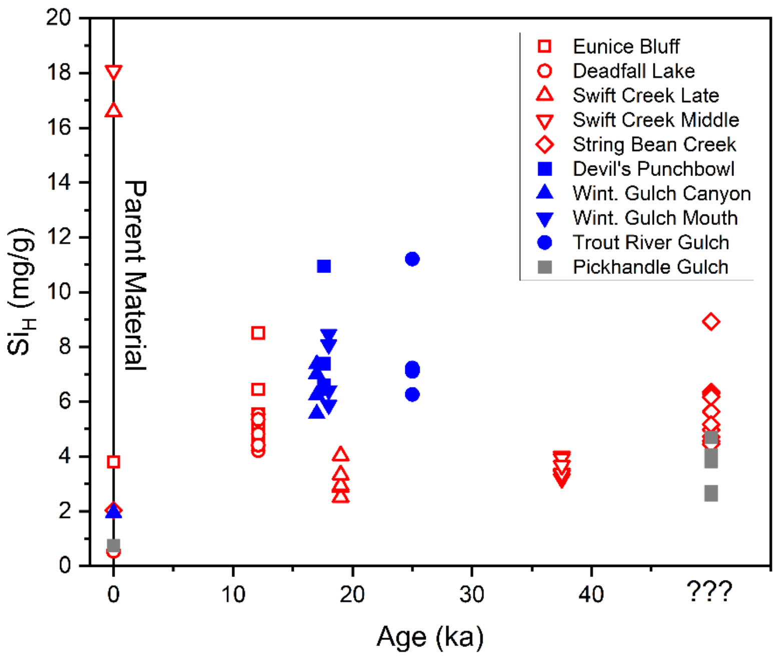

Figure S50 – Hydroxylamine Hydrochloride extractable Si (Si_H) plotted by soil pit age. Parent Material are given as points set at T = 0 ka.

S1.8 Dithionite Reducible Fe

The citrate dithionite extractant attacks both amorphous and crystalline Fe^{3+}-(oxyhydr)oxides, acting as a reducing agent at ~pH 7.3[21,24]. Fe^{3+} contained within oxyhydroxides and oxides (Fe_D) is more abundant in the wetter Klamath Mountains and Tablelands soils than within parent material (Figure S51). This increasing concentration of Fe^{3+} within the Klamath Mountains and Tablelands soils relative to parent material is reflected in large increases in Fe-concentrations in clay-size fraction material relative to parent material (Figure 3 in main text). The Klamath Mountain soils exhibit the largest increase in Fe-(oxyhydr)oxide abundance in relation to parent material of all examined field sites. At Pickhandle Gulch the increase in Fe-(oxyhdyr)oxide content is minimal compared to parent material values. Total Fe-(oxyhydr)oxide content appears relatively equal among the Tablelands and Klamath Mountain soils, with the exception of the 12.1 ka Eunice Bluff soil in the Klamath Mountains.

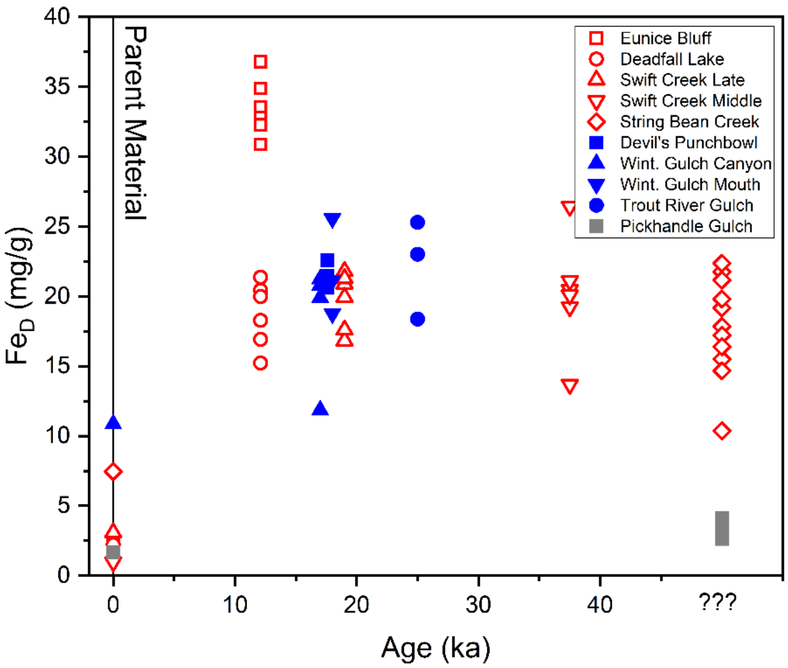

Figure S51 – Citrate Dithionite extractable Fe (Fe_D) plotted by soil pit age. Parent Material values are given as points set at T = 0 ka.

S1.9 Hydroxylamine and Dithionite Selective Dissolution Results Plotted by Depth

Total Fe in amorphous material measured by the hydroxylamine hydrochloride extraction (Fe_H) is separated into three non-overlapping groupings with Pickhandle Gulch soils exhibiting minimal amorphous Fe, Tablelands soils exhibiting the greatest total amorphous Fe, and the Klamath Mountains soils falling in-between (Figure S52). Total Fe in (oxyhydr)oxides measured by the citrate dithionite extraction (Fe_D) still shows Pickhandle Gulch soils possessing minimal total Fe content, while the Tablelands and Klamath Mountain soils are predominantly overlapping with one exception. Amorphous Si content measured by the hydroxylamine hydrochloride extraction (Si_H) is universally less abundant than extracted Fe though this likely reflects the selectivity of hydroxylamine hydrochloride for Fe[21]. Pickhandle Gulch soils on average exhibiting the most limited amorphous Si, Klamath Mountains soils intermediate values, and Tablelands soils possessing the greatest total abundances of amorphous Fe.

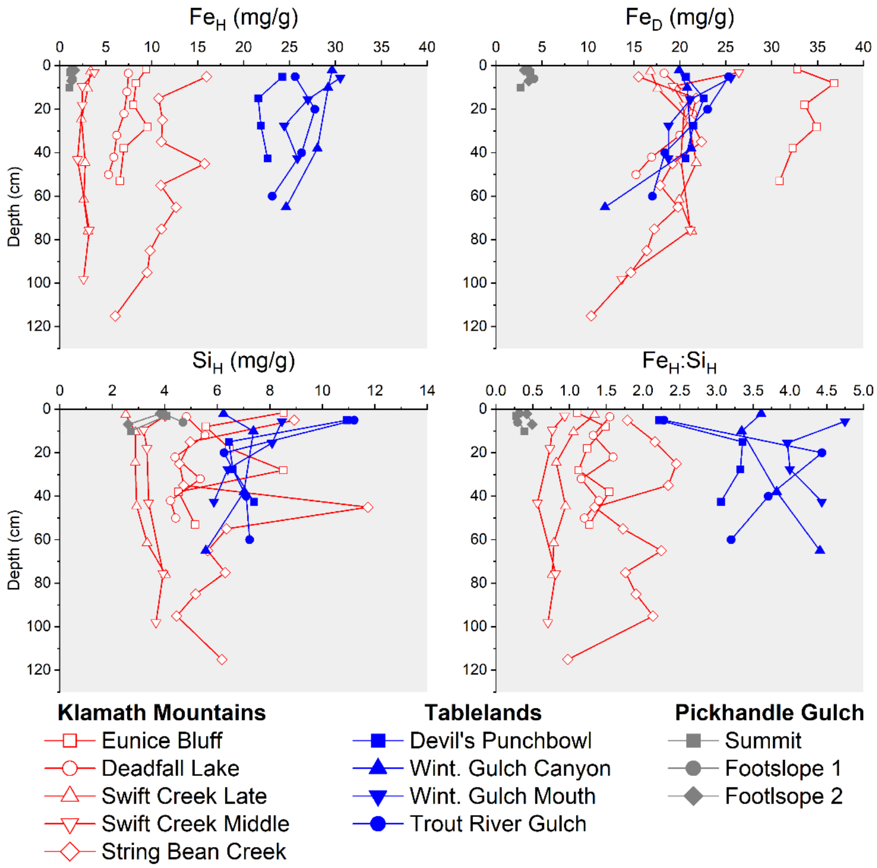

Figure S52 **–** Selective dissolution results plotted by depth for all examined soil pits from the Klamath Mountains, Tablelands, and at Pickhandle Gulch.

S1.10 Pyrophosphate Extractable Fe

Pyrophosphate extractable Fe (Fe_P), a potential measure of organically bound Fe[21,25], is uniformly minimal across all examined soils (Figure S47), indicating limited organic complexation of Fe. Fe_P values are slightly higher in the Klamath Mountains than within the Tablelands and exhibit a trend from higher surface concentrations to minimal concentrations at depth (Figure S53), consistent with the development of organically enriched A horizons in Klamath Mountain soils. Higher organic content in the Klamath Mountain soils likely results from pine needle litter from the prevalent pine forest cover[26]. The minimal Fe_P content in all examined soils suggests organic complexion does not exert a significant effect on Fe mobility or storage within the examined soils, but potentially exerts a larger effect on the Klamath Mountain soils than the Tablelands and Pickhandle Gulch soils which exhibit minimal vegetation cover.

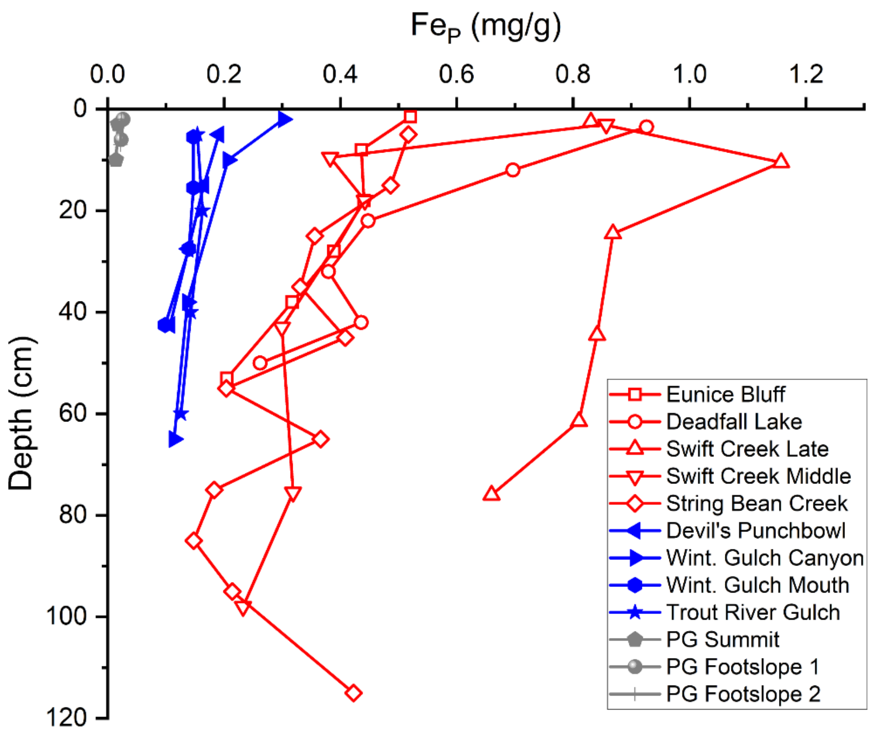

Figure S53 – Pyrophosphate Extractable (Fe_P) Fe plotted by depth for all sampled soil pits.

S1.11 Soil Field Descriptions, Soil pH, and Soil Loss on Ignition at 550 ^{o}C

Soil descriptions discussed here are given in Supplementary Data 3. Soil pH and loss on ignition at 550°C values discussed here are given in Appendix B.

Soil horizons are visible within the Klamath Mountains soils but soil differentiation appears much more limited within the Tablelands and Pickhandle Gulch soils. Initial development of transitional BC horizons was observed in the youngest Klamath soils, with reddened B horizons evident in the intermediate age Swift Creek soils, and a clay-film rich Bt horizon observed in the oldest String Bean Creek soil. Munsell color hue varies from 10YR to 7.5YR in the youngest Klamath soils (Eunice Bluff and Deadfall Lake) to up to 5YR in the oldest Klamath Mountain soil (String Bean Creek). By contrast, within the Tablelands soils Munsell color hue exhibits less reddening than in the Klamath Mountain soils, varying from exclusively 2.5Y in the youngest soil (Devil's Punchbowl) to exclusively 10YR in the oldest soil (Trout River Gulch). The limited color variation is consistent with a lack of B horizon development. A lack of visible accumulation of organic matter in the form of darkened surface material in Tablelands soils was consistent with a lack of A horizon formation. Pickhandle Gulch soils do not exhibit any significant color differentiation, with uniform 10YR hues. However, the surface horizons at Pickhandle Gulch did exhibit the development of vesicular features, indicating formation of incipient Av horizons.

Texture in the Klamath Mountains soils varied from loamy sands to sandy clays and sandy clay loams. In the Tablelands and at Pickhandle Gulch soil texture varied from sandy clay loams to loamy sands. Ped development was absent from all Tablelands soils, and, within the younger Klamath Mountain soils, was only observed at the Eunice Bluff site where small (<5 mm) sub-angular blocky peds were present. Greater ped development was observed within the older Klamath Mountain soils, with development of sub-angular blocky peds of up to roughly 5 cm in diameter in some soil horizons in both the String Bean Creek soil. Minor development of sub-angular blocky peds <5 mm in diameter was observed in the surficial vesicular horizons at Pickhandle Gulch.

Clay film development potentially attributable to secondary clay mineral development is absent in the youngest Klamath Mountain Soils (Eunice Bluff and Deadfall Lake) and progressively increases in the older soils. Very minor clay film development coating and bridging grains was observed in the Swift Creek Late soil, with more evident clay films coating and bridging grains within peds in the Swift Creek Middle soil. In the oldest Klamath Mountain soil (String Bean Creek) soil clay films coated and bridged grains within peds throughout the soil profile except for within the surface A horizon. Clay film development is absent from the soils of the Tablelands and at Pickhandle Gulch.

Loss on ignition values in the Klamath Mountains soils indicative of organic carbon content are highest at the surface and decline with depth in all soil profiles, reflecting the development of organically enriched A horizons. In the Tablelands, loss on ignition values do not change significantly with depth (6.2-11.0 wt.%), reflecting a relatively even distribution of organic carbon and the lack of well-developed organically enriched A horizons. Loss on ignition values from Pickhandle Gulch were very low (1.0 – 2.3 wt.%), reflecting limited organic carbon accumulation. Soil pH ranges were circumneutral in the Klamath Mountains (5.9 – 7.), slightly basic in the Tablelands (7.3 – 8.0), and more basic at Pickhandle Gulch (8.2 – 8.7).

S2 Supplementary References

1. Daniels, M. L., Anderson, S. & Whitlock, C. Vegetation and fire history since the Late Pleistocene from the Trinity Mountains, northwestern California, USA. *Holocene* (2005) doi:10.1191/0959683605hl878ra.
2. Sharp, R. P. Pleistocene Glaciation in the Trinity Alps of Northern California. *Am J Sci* **258**, 305–340 (1960).
3. Dickey, N. W. Chronology and Paleoclimate of Late Pleistocene Glaciation in The Klamath Mountains, CA. (Colorado State University, Northridge, 2016).
4. Osborn, G., Spooner, I., Gosse, J. & Clark, D. Alpine glacial geology of the Tablelands, Gros Morne National Park, Newfoundland. *Can J Earth Sci* **44**, 819–834 (2007).
5. Clark, P. U. *et al.* The Last Glacial Maximum. *Science* **325**, 710–714 (2009).
6. Baumeister, J. L. *et al.* Biogeochemical weathering of serpentinites: An examination of incipient dissolution affecting serpentine soil formation. *Applied Geochemistry* **54**, 74–84 (2015).
7. Harward, M. E., Carstea, D. D. & Sayegh, A. H. Properties of vermiculites and smectites: Expansion and collapse. *Clays Clay Miner* **16**, 437–447 (1969).
8. Bailey, S. W. Summary of recommendations of AIPEA nomenclature committee. *Clay Miner* **15**, 85–93 (1980).
9. Vali, H. & Hesse, R. Identification of vermiculite by transmission electron microscopy and X-ray diffraction. *Clay Miner* **27**, 185–192 (1992).
10. Sato, T., Watanabe, T. & Otsuka, R. Effects of layer charge, charge location, and energy change on expansion properties of dioctahedral smectites. *Clays Clay Miner* **40**, 103–113 (1992).
11. Moore, D. M. & Reynolds, R. C. *X-Ray Diffraction and the Identification and Analysis of Clay Minerals*. (Oxford University Press, New York, 1997).

12. Christidis, G. E. & Eberl, D. D. Determination of layer-charge characteristics of smectites. *Clays Clay Miner* **51**, 644–655 (2003).

13. Lee, B. D., Sears, S. K., Graham, R. C., Amrhein, C. & Vali, H. Secondary Mineral Genesis from Chlorite and Serpentine in an Ultramafic Soil Toposequence. *Soil Science Society of America Journal* **67**, 1309–1317 (2003).

14. Ulery, A. L. & Drees, R. L. *Methods of Soil Analysis: Mineralogical Methods. Part 5*. (ASA-CSSA-SSSA, 2008).

15. Mosser-Ruck, R., Devineau, K., Charpentier, D. & Cathelineau, M. Effects of ethylene glycol saturation protocols on XRD patterns: A critical review and discussion. *Clays Clay Miner* **53**, 631–638 (2005).

16. Poppe, L. J., Paskevich, V. F., Hathaway, J. C. & Blackwood, D. S. A Laboratory Manual For X-Ray Powder Diffraction. *US Geological Survey Open-File Report 1.041* 1–88 (2001).

17. Lévy, L. *et al.* Smectite quantification in hydrothermally altered volcanic rocks. *Geothermics* **85**, 101748 (2020).

18. Bergmann, J., Friedel, P. & Kleeberg, R. BGMN—a new fundamental parameters based Rietveld program for laboratory X-ray sources, its use in quantitative analysis and structure investigations. *IUCr Commision on Powder Diffraction Newsletter* **20**, 5–8 (1998).

19. Doebelin, N. & Kleeberg, R. Profex: A graphical user interface for the Rietveld refinement program BGMN. *J Appl Crystallogr* **48**, 1573–1580 (2015).

20. Peacock, S. M. Serpentinization and infiltration metasomatism in the Trinity peridotite, Klamath province, northern California: implications for subduction zones. *Contributions to Mineralogy and Petrology* **95**, 55–70 (1987).

21. Shang, C. & Zelazny, L. W. Selective Dissolution Techniques for Mineral Analysis of Soils and Sediments. in *Methods of Soil Analysis Part 5 - Mineralogical Methods* (eds. ULERY, A. L. &

RICHARD DREES, L.) 33–80 (Soil Science Society of America, 2008). doi:10.2136/sssabookser5.5.c3.

22. Ross, G. J., Wang, C. & Schuppli, P. A. Hydroxylamine and Ammonium Oxalate Solutions As Extractants For Iron and Aluminum From Soils. *Soil Science Society of America Journal* **49**, 783–785 (1985).

23. Chao, T. T. & Zhou, L. Extraction Techniques for Selective Dissolution of Amorphous Iron Oxides from Soils and Sediments. *Soil Science Society of America Journal* **47**, 225–232 (1983).

24. Holmgren, G. G. S. A Rapid Citrate-Dithionite Extractable Iron Procedure. *Soil Science Society of America Journal* **31**, 210–211 (1967).

25. McKeague, J. A. An Evaluation of 0.1 M Pyrophosphate and Pyrophosphate-Dithionite in Comparison With Oxalate as Extractancs of the Accumulation Products in Podzols and Some Other Soils. *Can J Soil Sci* **47**, 95–99 (1967).

26. Skinner, C. N., Taylor, A. H. & Agee, J. K. Klamath Mountains Bioregion. in *Fire in California's Ecosystems* 170–194 (University of California Press, 2006).
