# Supplementary material for: Fe-rich X-ray amorphous material records past climate and persistence of water on Mars
Source: Commun Earth Environ. 2024 Jul 7;5(1):364. doi: 10.1038/s43247-024-01495-4 (PMC11227439; doi:10.1038/s43247-024-01495-4)
Supplement: Supplementary file 3 — Description of Additional Supplementary Files [file 43247_2024_1495_MOESM3_ESM.pdf]

Description of Additional Supplementary Files

File name: Supplementary Data 1
Description: XRD data from powdered parent material, bulk soil, and clay-size fraction samples, as well as Rietveld fits of clay-size fraction samples.

File name: Supplementary Data 2
Description: Chemical data from bulk HF dissolution measured by inductively coupled plasma mass spectrometry and selective dissolutions measured by Atomic absorption spectroscopy.

File name: Supplementary Data 3
Description: Soil field descriptions and an analysis tracker showing which samples were processed for varying analytical techniques.
